# Moral disengagement and empathy in cyberbullying: how they are related in reflection activities about a serious game

**DOI:** 10.1186/s40359-024-01582-3

**Published:** 2024-03-21

**Authors:** Sofia Mateus Francisco, Paula Costa Ferreira, Ana Margarida Veiga Simão, Nádia Salgado Pereira

**Affiliations:** CICPSI, Faculdade de Psicologia, Universidade de Lisboa, Alameda da Universidade, Lisboa, 1649-013 Portugal

**Keywords:** Moral disengagement, Cyberbullying, Empathy, Adolescents, Intervention

## Abstract

Cyberbullying is a complex phenomenon with multiple factors involved, both contextual and individual factors, such as moral disengagement and empathy. This study investigated how moral disengagement and empathy could be related, longitudinally in cyberbullying events. Specifically, two gamified tasks (one for empathy and other for moral disengagement) were analyzed. These tasks were developed attending to the specificities of the cyberbullying scenarios presented in a serious game. To accomplish this goal, data from gamified tasks (*N* = 208), from 4 different moments, were analyzed through multilevel linear modeling. Results suggested that there was a change in adolescents’ moral disengagement over time. Participants with greater empathy revealed lower moral disengagement overall. Over time, adolescents with greater empathy revealed lower moral disengagement within their own growth rate. Overall, our results provide important information about the dynamic relationship between moral disengagement, empathy and cyberbullying, which informs future studies and interventions.

**Supplementary Information:**

The online version contains supplementary material available at 10.1186/s40359-024-01582-3.

## Introduction

Cyberbullying is a socially dynamic process [1], considering the overlap of roles in this type of aggressive behavior, since aggressors and victims are frequently also bystanders [2]. Moreover, despite the fact that an important role in cyberbullying situations is played by bystanders, either by joining in or stopping the phenomenon [3, 4], few interventions have focused on changing bystander behavior [5]. Thus, interventions to tackle cyberbullying are one of the main challenges to increase prosocial behavior online [6, 7], because cyberbullying is one of the major contributors to poor mental wellbeing [8]. These interventions have accompanied the technological advances, and serious games and gamified tasks have been incorporated, since they are educational tools that are effective, motivate students, and are efficient in raising awareness and changing attitudes of youth in several areas [9]. Also, Information and Communication Technologies (ICT) are able to promote emotional, psychological and social wellbeing to children and adolescents [10].

The relationship between moral disengagement (MD) and empathy in the context of bullying has already been studied by several investigations [11]. However, very few studies regarding interventions have considered the role of empathy and MD specifically attending to the online context, and with respect to cyberbullying [12]. Despite the innovative view of considering both constructs with respect to online contexts, to our knowledge there are no intervention programs focusing on these two constructs, and there is a need for evidence-based intervention strategies [11]. Therefore, in this investigation, the development and analysis of two gamified tasks embedded in a serious game are proposed, specifically aimed at examining empathy and moral (dis)engagement concerning cyberbullying incidents.

## Bystanders of cyberbullying

In both bullying and cyberbullying, bystanders are present, but their relevance is more critical in cyberbullying incidents due to their high number [12, 13]. They have the power to alter these incidents, not only in their course but also considering its’ effects [3]. Thus, bystanders may opt to intervene or not, which leads to different outcomes for those involved [3, 14]. Despite the potential to alter events, most bystanders remain passive [15], and low intentions to ask adults for help in these situations are also observed [16]. Passive bystanding is extremely important because the lack of intervention may be perceived by cyberbullies as an approval of their behavior [17]. This is why improving prosocial behavior of bystanders is so important. Additionally, previous experiences as cyberbullies are related to bystanders’ reinforcing behavior [18], whereas being a victim of bullying or cyberbullying was a positive predictor of helping the victim [4].

The bystander effect [19] has been one the most dominant paradigms to explain this bystander inaction [22], however, the Social Cognitive Theory (SCT) [20] may prove to be more useful, as it captures the complexity and contextualization needed in explaining cyberbullying dynamics [3]. For instance, bystanders who witness online aggression, with time may come to believe that it is normal and acceptable [21]. However, there may be a desensitization effect in terms of the decrease in empathic responsiveness towards victims, but not related to pro-cyberbullying attitudes [22]. Nonetheless, studies focusing on interventions have shown that bystanders’ empathy can be fostered, for example, through serious games [7].

### Serious games in cyberbullying

Serious games are considered an important educational tool for changing attitudes and raising awareness to different subjects, as they are effective and motivational for users [9]. Several serious games have already been created to intervene in bullying and/or cyberbullying, and they differ from each other, regarding features, objectives, and the targeted player age. A brief description of some games will be presented below, from the oldest to the more recent.

For example, the Cyberhero Mobile Safety Program was designed for children between 8 and 12 years of age and the six videogames included in the program teach children about digital well-being and citizenship [23]. Targeting different ages, the Bully Book was designed for young adults and adults (18–34 years old), and the goal is to help them practicing how to behave in case of a cyberbullying incident in a social network site [24]. Another resource designed for children is Monité, which was developed for children between 10 and 12 years of age to prevent bullying and cyberbullying behavior through didactic strategies that promote communication and collaboration among students, resilience and empathy [25]. For older children, Friendly ATTAC has two objectives, as it intends to increase positive bystander behavior and decrease negative bystander behavior among 8th grade students between 13 and 14 years old [5]. The Cooperative Cybereduca 2.0 is a videogame that takes place in an intervention, after the program Cyberprogram 2.0. This intervention aimed to educate adolescents about adequate use of ICT, as well as how to prevent and reduce bullying and cyberbullying situations [26]. Conectado was developed with the main goal of raising awareness of both bullying and cyberbullying in adolescents from 12 to 17 years of age [27]. Another resource is CyberBullet, which was developed to prevent online child abuse, in which the authors include cyberbullying, however they did not indicate the target age of participants [28]. Finally, NN – Lazarinis aims at augmenting children’s understanding of online risks, through several situations that might occur when going online [29].

As can be seen, serious games are an important tool to address cyberbullying, as they can include factors related to social environment and group interactions, such as social pressure [34] and friendship with other bystanders [19] that have been found to influence bystander behavior. Moreover, individual factors have been largely studied, first in relation to bullying and later, with respect to cyberbullying. For example, these factors include empathy [35], and MD [16], since they are present both in face-to-face and online interpersonal interactions [30]. Thus, considering this, it can be stated that how bystanders react to cyberbullying incidents is influenced by individual, contextual and social factors [31]. In our study, participants were placed as bystanders of cyberbullying, victims were social agents from the game Com@Viver, and this game provide participants opportunities to interact with the different social agents and decide how to intervene in different situations [7].

### Moral disengagement in cyberbullying

As Bandura proposed, “People suffer from the wrongs done to them regardless of how perpetrators justify their inhumane actions” [32, p. 101], thus distinguishing right from wrong is necessary, but not enough for moral conduct, because even when actions are justified, they are not free from negative effects. It is here that MD takes place. That is, the role of moral standards is to regulate action; however, behavior is not regulated in a fixed manner, as several psychological mechanisms function to disengage moral self-sanction from inhumane conduct [32].

MD mechanisms can be related to four different loci: behavior, agency, outcome and recipient. In the locus of behavior, *Moral Justification* refers to immoral conduct that is justified as serving social or moral intentions; *Euphemistic Labeling*, implies that by giving subtle names, the intention is to decrease the severity of the harmful behavior, and *Advantageous Comparison* is related to a contrast that is made with other behavior that are considered more wrong and severe. In the locus of agency, through the *Displacement of Responsibility* the perpetrator’s agentic role is minimized in detrimental conduct, because individuals view their actions as resulting from others’ orders, and through the *Diffusion of Responsibility* individuals tend to divide the responsibility for the behavior among a group. In the outcome of the behavior, through *Distortion of consequences* moral control is faded, since one’s conduct is overlooked, minimized, distorted or disregarded. Lastly, in the locus of the recipient, *Dehumanization* of the victims deprives them of their human qualities, and *Attribution of blame* to the victims, allows perpetrators to justify their behaviors because the victim is considered responsible [32–34].

MD is considered by some authors [21, 35–37] as an important risk factor for cyberbullying perpetration, especially from middle school to college education. Specifically, prior cybervictimization and cyberbystanding are related to subsequent cyberbullying perpetration, and it seems that MD is key in this relation, as it allows the aggression to continue by enabling individuals to justify their conduct and not feel remorse about their cyberbullying behavior [38]. Thus, it is important to gain a deeper understanding on how MD manifests in bystanders.

The relevance of MD in cyberbullying needs to be further investigated, especially regarding its trend of occurrence. MD may be stronger in the context of cyberbullying, than in bullying [39, 40], but some authors argue that the characteristics of the online environment may decrease the need to resort to these mechanisms [47].

Research concerning MD with respect to cyberbullying bystanders shows that higher MD attitudes of cognitive restructuring predicted negative bystander behavior, while lower MD attitudes of blaming the victim predicted positive bystander behavior [14]. Furthermore, in qualitative research several MD mechanisms were found to hinder aiding the victim, such as blaming the victim and distortion of consequences [41]. Nonetheless, adolescents predominantly perceive that cyberbystanders have the responsibility to morally engage when witnessing cyberbullying situations [41]. However, bystanders remain passive mostly, and this behavior is associated with higher levels of MD [42]. Specifically, several mechanisms have been known to be important in explaining passive bystander behavior, such as moral justification, diffusion and displacement of responsibility, distortion of consequences and attribution of blame [4, 43]. Despite this, bystanders can defend the victim, although the association between MD and defending behavior is not clear. Nonetheless, it is important to highlight that the type of defending behavior has not been specified [42]. Other authors found that constructive defending (a more prosocial type of defending) was negatively associated with MD, whereas aggressive defending was positively associated with MD [12, 44]. Moreover, besides defending or remaining passive, bystanders may reinforce cyberbullying behavior, and this has been positively associated with MD [42].

With respect to longitudinal studies, the relationship between MD and cyberbullying has been analyzed by few. A recent systematic review [45] regarding the relationship between MD and bullying (also including cyberbullying) longitudinally among children and adolescents, found only four studies that included cyberbullying behavior. Additionally, none of the studies analyzed have focused on bystanders, but on cyberperpetration and cybervictimization. Nonetheless, they are important to understand how MD can be related to perpetrating the cyberbullying cycle, we will only mention one, as it included both online empathy and MD regarding virtual contexts. Thus, Marín-López et al. [46] was the first and only study so far to include the relationship between online empathy and MD through technology regarding cyberbullying. The authors indicated that higher cybervictimization was related to higher levels of both online empathy, as well as MD, and higher cyberperpetration was related to higher levels of MD. This study analyzed the same two variables as our investigation; however our focus is on bystanders’ behavior.

Despite the studies mentioned above, none have focused on the relationship between MD and empathy in cyberbullying, considering a longitudinal design, where participants are placed as bystanders of cyberbullying situations and with objective measures, such as in-task performance in different scenarios.

Hence, MD mechanisms are important to consider when designing prevention and intervention programs [47] to tackle cyberbullying. Thus, concerning MD in cyberbullying incidents, we propose to answer the following research questions:

1. Is there change in adolescents’ MD regarding cyberbullying situations over time?
2. If so, is this a linear change or a more complex trend?

### Empathy in cyberbullying

Empathy is a complex psychological phenomenon of key importance in social interaction [48], which plays an important role in moral development [49], including in emotions, attitudes and behavior [50]. Empathy can be viewed as both as emotional and a cognitive response to other people’s situations and refers to the ability to understand and feel the emotions of others [51]. It is considered a multidimensional construct, as it includes affective and cognitive empathy. While affective empathy refers to the ability to experience other’s emotions, cognitive empathy respects to the ability to understand the situation, as well as the perspective of others [52].

Although empathy can be considered a relatively stable trait [59], implying that some persons are more empathic than others [53], there is also a widespread consensus that empathy is predetermined by circumstances [54]. Thus, empathy is better conceptualized as something that is target-related and associated to the complexity of interactions (i.e., characteristics of the target and the situation that is occurring), and empathizer-related (i.e., traits, experiences and motivations), which are integrated in a cultural context [55].

Empathy is extremely relevant in cyberbullying situations, as it has a role in regulating bystanders’ prosocial behavior online [56], and can be considered a protective factor [57]. For example, individuals who have higher empathic concern are more likely to help cyberbullying victims, whether; individuals with lower levels are more prone to continue cyberbullying aggressions, or to remain passive when observing these situations [4]. It has been argued by other authors [56] that the activation of cognitive empathy tends to increase the likelihood of bystanders to intervene in cyberbullying situations, but the same does not occur with respect to affective empathy.

Moreover, empathic response from cyberbystanders has been found to vary according to the severity of the cyberbullying incident, and thus, when empathy is higher, the likelihood of bystander intervention is also higher [58]. Additionally, when the cyberbully is very different from the victim, the effect of the incident severity on the willingness to intervene is greater than when both are more similar [58]. Moreover, Huang et al. [59] found that the severity of the cyberbullying situation was associated with the intention to intervene. Moreover, feelings of personal responsibility for intervening increased as the severity also increased, which also increased the intention to intervene. And finally, on one hand, when bystanders presented low empathy, they were more prone to intervene when the incident was more severe. On the other hand, when bystanders presented high empathy, they were inclined to intervene independently of the severity of the situation [59].

In view of this, empathy is considered an important construct to be included in programs that aim to increase bystander intervention [3, 15]. Also, digital games might be included in these programs, as they are relevant tools to help foster empathy [6, 9].

This study will consider the emotional and cognitive components of empathy. On the emotional side, three components commonly studied will be analyzed. The first is *feeling the same* emotion as the other person, the second is *personal distress*, which refers to feeling of distress when someone perceives others’ pain; and third is *feeling compassion* (or *empathic concern*) for another person. On the cognitive side, we have cognitive empathy (or empathic accuracy) that refers to how well an individual can perceive and understand the emotions of another [55].

In previous research [60], we used the perspective of Baron-Cohen and Wheelwright [61], which combine both cognitive and affective components, as mentioned above. However, from a practical perspective (as will be discussed in the instrument section), we felt the need to differentiate according to different dimensions of the emotional empathy, which is not explicit in Baron-Cohen and Wheelwright’s [61] work.

### Relationship between empathy and moral disengagement in cyberbullying

Empathy has been linked to MD in several studies. Research on why people offer or omit help when explicitly asked for assistance found that both empathy and personal distress affected MD [62]. That is, the activation of these mechanisms was prevented by empathy, whereas it was fostered by personal distress. In cyberbullying the explicit call for help may not exist, however, empathy and MD are important in explaining cyberbystanders’ behavior [35, 68]. It is known that cognitive costs, such as effort and inefficacy can deter empathy [49]. Thus, considering the above, we argue that combining both empathy and MD training in cyberbullying interventions, may facilitate feeling empathy, as participants may become conscious of the automatic use of MD mechanisms, and understand that it is important to be more empathic in these situations and that their intervention matters - which may, in turn, reduce the feelings of effort and inefficacy.

Some studies consider that the moral component of active help is of particular importance when designing bullying and cyberbullying interventions [63]. In particular, it is considered by these authors that active help includes empathy, as well as the personal responsibility for intervening, which may be related to MD mechanisms, such as diffusion and displacement of responsibility. Furthermore, other studies go beyond this, and found that specific mechanisms of MD mediated the relationship between bullying and empathy. Specifically, minimization of responsibility, distortion of consequences and dehumanization mediated the relation between bullying perpetration and cognitive empathy, whereas cognitive restructuring and the distortion of consequences mediated the relation between bullying perpetration and affective empathy [64].

Considering empathy and MD in online contexts, the only study to date that examined this relation found that higher levels of MD were related to higher levels of online empathy [46]. These results were contradictory to other studies considering earlier investigation on both bullying and cyberbullying, as discussed previously. The same authors also found that higher cybervictimization was related to higher levels of MD through technology.

Many studies related empathy and MD, however, their focus has been mainly on bullying and cyberbullying perpetration [70, 71]. Moreover, few studies have analyzed the relationship between online empathy and MD through technology [12]; nonetheless, none have done this with respect to objective measures [65], as well as considering a longitudinal design with respect to an intervention program and concerning bystanders’ intervention. Thus, to fill this gap, we proposed to analyze MD specifically with respect to cyberbullying scenarios and empathy in virtual contexts, considering bystanders. Therefore, we propose to answer three more research questions in the context of cyberbullying situations presented previously in the game:

3) Is adolescents’ empathy regarding the cyberbullying scenarios related to their MD?
4) Do adolescents with greater empathy with respect to the cyberbullying scenarios reveal lower MD overall?
5) Considering the cyberbullying scenarios presented, are there any differences within and between individuals in their MD over time related to their empathy?

## Method

### Design and procedures

In a previous larger study, a quasi-experimental design was conducted, with control and experimental groups. The control group solely responded to questionnaires over time, without receiving any additional interventions, while the experimental groups were included in a program [2], in which the activities described in this study were part. In this investigation, the results of the experimental group will be analyzed.

This study presents a longitudinal design, while exploring the relationship between MD and empathy, through two gamified tasks specifically developed for this study. After playing 4 sessions (approximately 50 min each) of the game Com@Viver, which yielded 4 different cyberbullying situations, students played 4 sessions of the gamified tasks. They were allowed to think about their behavior as a bystander while answering several activities. These activities were conducted once a week for 4 weeks.

### Participants

A total of 208 students (*M*_age_= 13.15, *SD* = 1.21, 54.8% males), 39% from the 7th grade, 24% from the 8th grade and 37% from the 9th grade participated in this study. Considering each school year separately, in the 7th grade the mean age was 12.34 years old, 51.2% were boys, 90% of whom were Portuguese, 5% were from African countries (e.g., São Tomé and Príncipe, Angola, Cape Verde), 1 student was from Spain and other from Brazil. With respect to the 8th grade, the mean age was 12.98, 56% of whom were boys, and 92% were Portuguese, 2 students were from African countries (e.g., Angola and Mozambique) and 2 other students had dual nationality (Italian-Brazilian and Portuguese-Spanish). Finally, from the 9th grade, the mean age was 14.14, 57.1% of whom were boys, 84% were Portuguese, 5% were from African countries (e.g., Angola, Cape Verde, Guinea, Mozambique), 6.5% were from North America and South America (e.g., Brazil and Venezuela) and 2 students were from Eastern European (e.g., Ukraine and Moldavia).

### Resources and instruments

#### OPT2Bgood program

For this investigation, a program entitled OPT2B good was implemented, which consists of two related components: the serious game Com@Viver and the gamified tasks about the game sessions. In this work, only the results of the tasks related to empathy and MD will be analyzed, however, both resources are related to each other. Thus, the game will be briefly described. In this investigation, the game Com@Viver is used only for contextualization, as it is part of a more extensive anti-cyberbullying program designed for adolescents [6] and which integrates the quasi-experimental study referred above.

#### Com@Viver serious game

Com@Viver is a multiplayer serious game designed to study bystanders’ reactions to cyberbullying incidents and to foster empathy, with the broader objective of promoting prosocial behavior [6]. Com@Viver was designed as a representation of a Social Networking Site (SNS) [74], since they are of great importance in youth socialization [66]. The SNS is populated by 12 Social Agents (SA) that represent school students, and they are able to comment on posts in the feed and on the chat, according to their roles (i.e., aggressors, victims and bystanders). The players (i.e., real participants) always have the role of bystanders [67].

The player’s goal is to organize a school trip, with a limited number of participants allowed; therefore, only the 3 most voted groups can go on the trip. Thus, the players’ group is formed by 3 students and the 12 SA formed the other 4 groups. The players interact in the Com@Viver social network by liking or disliking posts and commenting on these posts. As with many serious games, players may also interact to an SA [9] in the chat [6], which allows them to reflect about the incident. In every session, the player will witness a cyberbullying incident, presented by different scenarios [9], where one of the SA, the aggressor, will make a public post targeting one or more victims. Although these situations are fictional, they were based on real stories and on language used by adolescents’ discourse online [68]. It is the way players react to cyberbullying incidents that counts for the final score in each session. That is, when observing the incident, multiple possibilities of reactions are allowed, both in the comment section and the chat, and these responses directly affect the player’s score, considering their negative or prosocial nature [69].

#### Gamified tasks

The other component of the intervention is the reflection activities, which were created in order to help students take conscious about their opinions/beliefs about cyberbullying, from the beginning of the situation (i.e., motives) until the end of the episode (i.e., consequences for the victims). These activities are like mini-quizzes, where participants can give their opinion considering the above mentioned, which allows us to understand how adolescents would react to each cyberbullying incident.

In this investigation, we are going to analyze a task related to MD and another related to empathy. Thus, the gamified tasks begin by helping the student remember his/her intervention during the session of the game Com@Viver (Appendix B, Figure B.1). With respect to the MD task (Appendix B, Figure B.2 and B.3), the specificities of each cyberbullying incident and those involved were conveyed. In this activity, a sentence appeared as the participant responded to the previous sentence. The content of all 17 sentences was based on interviews conducted with adolescents regarding cyberbullying fictitious scenarios (see Appendix A, Figure A1 to A4) and the respective content analysis based on the SCT [34]. These sentences (e.g., “I think Helder published the post to joke around with Abel.”) were related to all the MD mechanisms, and reflected the specificities of each cyberbullying situation (i.e., name of victim, story behind the situation, etc.). MD was measured from 1 to 5 on all items in the database. For example, 1 = “Totally disagree” and 5 = “Totally agree”, thus participants had to assign a value in order to give their opinion about that situation. This task was completed by participants at 4 different moments, as was done with the empathy activity.

With respect to the Online empathy task (Appendix B, Figure B.4 and B.5), the purpose was that the participant could have the opportunity to support the victim. The task is conveyed on a mobile phone from where the participant could send a message (several or none) according to what they were feeling with respect to that cyberbullying case (e.g., “I understand what you must be feeling.”). Participants had to assign a value from 1 to 4 (a bit to a lot), in order to give him/her opinion about that situation. Eight messages were available (Appendix B, Figure B.4 and B.5) and represent cognitive empathy and emotional empathy (including its subcomponents: feeling the same, personal distress and empathic concern).

Considering tasks that try to induce behavioral change through simulations, the transfer to reality of what participants learn, sometimes is not obvious, but can be achieved through debriefing [70], since it allows to reflect on the in-game performance [71]. The gamified task activities are much like serious games, in the sense that participants choose their path along the way, however in a simpler way, as the activities are more or less like mini-quizzes. Nonetheless, like in serious games, these tasks allowed us to collect data during play, which is processed and stored during the task, providing material for both in-game debriefing and end-of-game debriefing [72], from session 2 to 4.

According to the description above, feedback for the empathy gamified task was provided at the end of the sessions (e.g., “It’s good to know that we are understood. Thanks for your support.”, Appendix B, Figure B9), and the MD gamified task had feedback both at the end of the activity (e.g., “I think Helder published the post to joke around with Abel.” “You answered totally disagree. It is true because jokes should not hurt.”, Appendix B, Figure B3) and at the end of the sessions (e.g., “Take responsibility.”, Appendix B, Figure B7), because the latter was longer and therefore, we considered that two types of feedback would improve its efficacy. For the final debriefing, a time-oriented visualization was used (Appendix B, Figures B6 to B11) that puts the focus on the time of occurrence of the interactions by using a time line of events [70]. This organizes information, according to the same order of the activities, and players click along the timeline to obtain information about the activities. By clicking, the content of the interaction (i.e., their performance in the specific activity) can be seen, and a feedback message appears.

Both tasks were evaluated with the Item Response Theory (IRT) with the Winsteps software program by Linacre [73] through Rasch analysis polytomous methodology to confirm the unidimensionality of each resource and to examine participants’ scores of MD in cyberbullying situations and online empathy. Participants’ scores were estimated on a one-dimensional logit scale and evaluated the properties of both tasks. An analysis was conducted for each task and for all occasions.

All items were assessed to understand if they had excessive infit and/or outfit mean square residuals. All items showed infit/outfit scores lower than 1.5, as well as 𝑧 statistic > 2.00, as recommended in the literature [74] with the exception of items 17 and 6 for MD throughout all four sessions and item 7 for Online empathy in sessions 0 through 2 and item 5 in session 2. Nonetheless, the Item Separation Reliability presented good scores throughout all four sessions for MD (0.97) and Online empathy (0.99, 0.98, 0.98 and 0.98, respectively), as well as reasonable scores for Person Separation Reliability (PSR) (MD: 0.86 in all sessions; Online empathy: 0.77, 0.72, 0.75 and 0.73, with non-extreme person values, respectively), as indicated in the literature [75]. We considered other reliability indicators from the Rasch measures such as the Cronbach’s alpha, which revealed good scores for the MD task (𝛼 = 0.91, consistently) and for the Online empathy task (𝛼 = 0.80, consistently) in all sessions. The scores indicated good internal consistency [76] although the PSR revealed some difficulty on the participants’ behalf. The distribution revealed a reasonable range of difficulty for the MD task (− 1.59 <𝐷𝑖< 1.09 consistently throughout all four sessions) and for the Online empathy task (− 0.63 <𝐷𝑖< 0.85; −0.64 <𝐷𝑖< 0.79; −0.74 <𝐷𝑖< 0.78; −0.63 <𝐷𝑖< 0.84, respectively).

### Procedure

For this study, authorization was requested and granted by the Ministry of Education of Portugal, the Portuguese National Commission of Data Protection, the Deontology Committee of the Faculty of Psychology of the University of Lisbon, schools’ boards of directors, teachers, parents, and adolescent participants. Participants completed the gamified tasks in a classroom context with computers with Internet access in their own schools, accompanied by researchers of this study. Before performing the tasks, all students were informed that their participation was confidential, anonymous and that they could quit at any time they wanted to. Moreover, they were informed that they could have psychological support (i.e., with a professional psychologist) if they needed to talk to someone during or after participating. All students questioned chose to participate in the study.

### Data Analysis

***Process Data.*** Responses from the participants in the MD task were used, where they revealed their level of MD regarding the cyberbullying situations throughout the sessions as our process data. Then, all item responses were aggregated by the dimension of MD, as indicated by Item Response Theory. The aggregation was done by day to obtain a mean score for the group of individuals of MD for each day of the training.

Multilevel Linear Modeling (IBM, SPSS, 22.0) was performed for repeated measures designs to measure the differences in MD within and between individuals. For the analysis, a sample size of 832 response entries (4 response entries per participant) was used for MD at level 1 and of 208 participants at level 2. Participants’ responses were measured on four occasions.

MD was the dependent variable, whereas time and empathy were considered the predictors. Data was structured at the within-person in time level (level 1) and the between person level (level 2). We used Restricted Maximum Likelihood estimation for all analyses, since it is a technique which enables unbiased estimation of the variance components with smaller samples [77]. The variables were introduced in SPSS in three steps to test any interaction effects.

In a first step, an intercept-only model was computed to determine the variability present in MD at each level [78]. In a second step, we proposed to define the shape of the growth trajectory. A model including a linear time variable only and another with orthogonal polynomials was tested, which did not yield any significant results in explaining individual growth in MD. The model with linear time and a quadratic time variable yielded significant results. Hence, the quadratic trend [78] was chosen. In a third step, to understand whether empathy was related to different growth patterns of MD, differences in development within and between individuals were studied. Specifically, the purpose was to understand if there were differences within the same individuals and between different individuals with regards MD over time considering empathy levels. To understand if these differences existed over time, the level 1 model with time specified as both linear and quadratic to describe individuals’ growth over time were combined, assuming the intercept varied between subjects and that the time slope was randomly varying [78]. Presenting a parsimonious model was the focus here.

The improvement of the models over the previous one was assessed with the corresponding likelihood ratios. The difference in likelihood approximates is in accordance with the chi-square distribution (i.e., change in degrees of freedom between models by subtracting the number of new parameters added to the model from the parameters of the previous model). Therefore, the differences in the deviances (by subtraction) are reported as evidence that the model with the covariates fits the data better than the model with the intercept and time, as well as the intercept-only model.

## Results

Firstly, means, standard deviations and correlations of each variable were computed (Table 1).

**Table 1:** Descriptive statistics and correlations of variables for multilevel analysis

Variables	Correlations		Level 1 (*N* = 832)				Level 2 (*N* = 208)
	1	2	0	1	2	3	
1. Empathy		− 0.36**	1.45(1.00)	1.41(0.99)	1.25(0.96)	1.10(0.93)	1.31(0.98)
2. Moral Disengagement	− 0.36**		1.86(0.63)	1.85(0.72)	1.79(0.71)	1.96(78)	1.86(0.71)

Note.^†^*p* < 0.10;^*^p < 0.05; ^**^p < 0.01. Day-level correlations are below the diagonal (*N* = 832). Person-level correlations are above the diagonal (*N* = 208). The Level 1 means and standard deviations are reported according to the time variable (from 0 to 3). Level 2 is reported on the right as the total mean (and standard deviation) of the participants

In the null model (Table 2), at level 1, the estimates for variability in the average individual’s MD was 0.22 around his/her own true growth trajectory [79]. At level 2, the variance was 0.29 (*Wald Z* = 8.38, *p* < 0.001), which suggests there was sufficient variation in intercepts across individuals. The proportion of variance in MD within individuals was 43.4% and between individuals was 56.6% in this model. Hence, there was change in adolescents’ MD over time.

Model two focused on defining the shape of adolescents’ growth trajectory and on defining whether the intercept and slopes varied across individuals. From the fixed effects (level 1), both linear (*β* = − 0.135; *p* < 0.05) and quadratic (*β* = 0.05; *p* < 0.01) polynomials revealed to be significant in explaining adolescents’ growth in MD, suggesting that both should be used in the following analyses. These results provided evidence that adolescents’ own change in MD was quadratic throughout time. From the results at level 2, which determined whether time-related slopes were randomly varying across individuals, we concluded that the proportion of variance in MD was 43% within individuals and 57% between individuals in this model.

In model three, adolescents’ empathy and the relationship with their MD were examined. The intercept (*β* = 2.086; *p* < 0.001) in this model was the MD true grand mean for individuals who revealed less empathy. Also, adolescents’ empathy was significantly related to their MD (*p* < 0.001). The coefficient for empathy (*β* = − 0.136) revealed that adolescents with greater empathy had an estimated MD grand mean of (*β* = 1.95), which was lower than the intercept of students with lower empathy, thus, suggesting that those with greater empathy revealed lower MD overall. This shows that adolescents’ empathy was related with their average MD. Differences within and between individuals’ growth rates in MD related to empathy were also examined. Model three revealed no significant differences between individuals in adolescents’ MD growth rates related to empathy. However, there were significant differences within individuals in adolescents’ growth rates of MD regarding empathy (*β* = − 0.034; *p* < 0.05). This suggests that over time, adolescents with greater empathy revealed lower MD within their own growth rate.

The model with the covariates revealed an improvement over the intercept-only model and the intercept + time model (MD: Δ *deviance* = 19.61, *df* = 2, *p* < 0.01; Δ *deviance* = 20.49, *df* = 5, *p* < 0.01).

**Table 2:** *Fixed and random effects parameter estimates for models predicting moral disengagement*

Parameters		Moral Disengagement	
	Intercept-only	Intercept + Time	With Predictors
*Fixed Effects*			
Intercept	1.86**(0.04)	1.88**(0.04)	2.08**(0.07)
Time		-0.13*(0.05)	-0.27**(0.08)
Quadratic Time		0.05**(0.01)	0.09**(0.02)
Empathy			-0.13**(0.03)
Empathy*Time			0.09^†^(0.05)
Empathy*Quadratic Time			-0.03*(0.01)
*Random Effects*			
Repeated measures	0.22**(0.01)	0.21**(0.01)	0.18**(0.01)
Intercept	0.29**(0.03)	0.29**(0.03)	0.23**(0.03)
Time			0.01(0.00)
Quadratic Time			0.00(0.00)
*Deviance*	1420.19	1421.07	1400.58
AIC	1424.19	1425.07	1408.58
BIC	1433.52	1434.40	1427.22

Note: Standard errors are in brackets. ^†^*p* < 0.10;^*^p < 0.05; ^**^p < 0.01

## Discussion

This study investigated how MD and empathy could be related, longitudinally in cyberbullying events. Accordingly, MD changed over time, and a relationship between empathy and MD in adolescents’ responses to the gamified activities was identified. Specifically, adolescents with greater empathy revealed lower MD overall.

Considering the type of data presented in this study, it is important to highlight that bystanders’ emotional and moral involvement with cyberbullying needs to be contextualized [16], and therefore, data presented will be more accurate with this type of objective data task, as opposed to using questionnaires. In terms of the empathy activity, it reflects diverse behavior and content (e.g., send a message or not to the victim and the type of message selected), which seems more accurate to measure empathy in terms of action, and not only intention. Specific results will be discussed, according to the order of the research questions.

This study found that not only was there a change in MD over time, but this change is quadratic. We may hypothesize that this quadratic trend may be due to the specificities of each cyberbullying incident. That is, context is central in the selectivity of MD, as the assessment of the situation guides subsequent behavior [80]. Thus, the different characteristics of the situation, along with the different sex of the aggressor and the victim from the hypothetical cyberbullying scenarios,might explain this trend.

For example, the lowest score on MD (1.79) was observed in the third scenario, and the highest score (1.96) was recorded in the fourth scenario. With respect to the properties of the incident, the content of the third case (“I thought you were gay Samuel! After all, you fell in love with the biggest waste of oxygen out there”) is related to insults and some sort of making fun, or may be considered verbal violence, which are two of the most common cyberbullying acts [57, 81]. Considering the high frequency of these acts, adolescents generally tend to believe that it is very common and less serious [82], and probably they do not feel the need to engage in MD mechanisms as much. However, the fourth case (“Abel, I haven’t forgotten what you said of Tatiana. I have already told you! I’m gone share that photo of you with Patrícia!”) is a threat related to outing or disclosing someone’s personal information which is less common [57, 81], and it has a greater impact on victims. Thus, it could be considered more serious [82]. In this type of cyberbullying acts, it is expected that MD mechanisms come into play, considering the seriousness of the situation and the fact that it might raise more feelings of guilt, for example, for not intervening.

Usually, when incidents are perceived as more serious, bystanders are more willing to help the victims [17, 83], and this would also decrease the tendency to blame the victim [83], which is one of the moral mechanisms used to explain bystanders aggressive behavior towards the victim [2]. Thus, we might argue that the fourth scenario is the one which could lead to less victim blaming, considering that it seems to be more severe [83] than the third scenario, and consequently, it would imply less MD scores. Nonetheless, the use of MD is higher. This would not be expected considering Bastiaensens et al. [17], however, we believe that the reason is related to the fact that the aggressor belongs to the ingroup, and therefore, bystanders would act differently towards the victim. In this case, the fact that the aggressor is from the ingroup might mediate the use of MD mechanisms. This may be possible because attitudes of prejudice and behavior of discrimination are generally against members of the outgroup [84], therefore what members of the ingroup do is more acceptable.

Therefore, the discrepancy referred before might be explained by group belonging [84, 85], because we have the tendency to classify groups according to our belonging, thus, we can consider as “we” (ingroups) and as “they” (outgroups) [84]. For example, in case 3, the victim belonged to the players’ ingroup and the aggressor belonged to the players’ outgroup; and in case 4, the victim belonged to the players’ outgroup and the aggressor to the players’ ingroup [7]. That is, when the victim belonged to the ingroup (3rd scenario), participants might have felt more responsible to protect him/her, and therefore, would use less MD mechanisms in this situation. This might also be related to participants feeling more empathy and intention to help the victim when he or she was from the ingroup [85]. Also, although the tasks from this study were based on a serious game, a parallel of feelings similar to friendship might have arisen, which could also explain these results, as friends tend to help each other [41]. Furthermore, empathizing with the victim is the driving force for individuals to want to help and support the victim [41], which consequently would decrease their use of MD mechanisms.

When the victim is from the outgroup (4th scenario), participants would probably feel less responsible for his/her wellbeing [7]. Also, these results are in line with previous qualitative research that found that not knowing the victim is one of the main reasons to not taking action in cyberbullying situations, and in some way, it can be considered a sort of displacement of responsibility [4]. Thus, having this in mind, participants would probably use more MD mechanisms in order to decrease their guilt and shame, for not taking responsibility in a situation, when otherwise they would act prosocially.

Considering the aggressor of both scenarios, in the third scenario, the aggressor is from the outgroup, which would make participants judge more, whereas when the aggressor belongs to the ingroup (4th scenario), players might assess the situation as being less serious [7], therefore, this would increase their use of MD mechanisms. These results seem to agree with previous research that found that when there is a friendship between bystander and aggressor, bystanders will tend to be passive, or join in on the aggressive behavior [86], and because of that they would probably use more MD mechanisms.

When comparing the third and fourth scenarios, it can also be observed that the sex of the aggressor is not the same. In the third, the aggressor is a girl, while in the fourth the aggressor is a boy. Considering that boys are more likely to cyberbully others [87], the fact that in the third scenario the aggressor is a girl might lead participants to not take that situation as serious as the fourth scenario, perhaps because aggressive behavior would not have been expected from a girl, and the situation would not have been evaluated as a cyberbullying event.

Considering the first and second scenarios, which had almost the same MD mean, it can be argued that in this case, the ingroup and outgroup perspective might not explain the results. In the first case, both aggressor and victim are from the outgroup, and in the second case, both are from the ingroup. While being from the outgroup might lead to a decrease in the use of MD mechanisms, this is not the case with lower mean. The relatively high mean might be explained by the type of aggression. In this case, (“Tatiana you moron! You’re ugly and fat! Don’t even think that you’re going to the trip! LOL!”), several types of aggression can be considered, such as, insults, making fun, sharing images without authorization [81] and social exclusion. Despite both being from the outgroup, which generally leads to a lesser use of MD mechanisms [7], the fact that this cyberbullying incident includes sharing images that is considered as being the most hurtful [88], and less frequent [81] which might lead to a higher use of these mechanisms.

Concerning the relation between empathy and MD, which was our third research question, it is not surprising to see that both are related, as these results corroborate the findings from Haddock and Jimerson [89], who found that adolescents involved in bullying scored higher in MD and tended to score lower in empathy. Also, Bussey et al., [90] found that the relationship between MD and aggression was moderated by empathic concern and perspective-taking. Thus, this may explain why higher levels of empathy were related to lower levels of MD in bystanders.

According to Van Cleemput et al. [4], lower levels of empathy were observed in adolescents who participated in cyberbullying situations. Thus, this might explain our results, as bystanders with diminished empathic skills could potentially exhibit higher MD scores, given that MD is identified as one of the factors contributing to an increased likelihood of aggressive behavior [91]. That is, both trait levels of empathy, as well as situation-specific empathy for those involved appear to predict bystander behavior, indicating that the reactions bystanders demonstrate can also be influenced by situational factors that are related to cyberbullying incidents, per se [92]. In this study, the serious game Com@Viver provided us with a context to analyze this variable, as it included four different cyberbullying cases (Appendix A, Figures A.1 to A.4), with distinct characteristics and participants [6, 7].

Finally, concerning the fourth and fifth research questions, adolescents with greater empathy revealed lower MD overall, and particularly adolescents with greater empathy revealed lower MD within their own growth rate over time. To our knowledge no studies have analyzed how MD evolves throughout time, and in relation to empathy, considering the specificities of the online and cyberbullying contexts, with respect to bystanders’ intervention. In fact, as discussed previously, Falla et al [64] considered that the increase of MD mechanisms would be related to lower levels of affective and cognitive empathy, with respect to bullying perpetration. For example, they found that cognitive restructuring and distortion of consequences, mediated the relationship between bullying and affective empathy. The latter also mediated cognitive empathy. Despite these results from previous studies referring to bullying, our research complements these findings considering that the more use of MD mechanisms seem to be related to less empathy scores throughout time. However, empathy online and MD through technology were investigated as longitudinal predictors of cybervictimization and cyberperpetration by Marín-Lopez et al. [46], and a higher level of MD was found to be related to a higher level of online empathy, which was unanticipated. These results contradict ours, nonetheless, our results refer to bystanders’, and therefore, there may be differences according to the role that is displayed in the cyberbullying cycle. Moreover, further research is needed in order to clarify the relationship between empathy in virtual contexts and MD with respect to cyberbullying. In fact, Nasaescu et al. [93] found that dehumanization and attribution of blame predicted stability of the high bullying victimization pattern over time. This highlights the need to work on MD over time, considering not only victims, but the fact that there are overlapping roles, as discussed before. Furthermore, our study showed that it is possible to develop empathy through the use of serious games, as Ferreira et al. [6] concluded, as well as through the use of gamified tasks. Furthermore, this study contributes to the literature in the sense that it integrates the awareness of the use of MD mechanisms through this recent technologic approach, in order to improve prosocial behavior online.

With respect to interventions that include MD components, some authors have found promising results. For example, Cross et al. [94] found that the Cyber Friendly Schools Program was associated with a significantly higher decline in the probability of involvement in cybervictimization and cyberperpetration from pre to the first post-test. Despite not being particularly described in the intervention, MD is referred to as one of the risk factors integrated into the social-ecological framework of the program [94, 95].

Moreover, Barkoukis et al. [96] found that the intervention group had significantly lower scores in MD, than students in the control group, however, empathy levels and other social cognitive variables were not statistically significant. Furthermore, the Bullying Literature Project, including targeted lessons to discourage MD, was successful in decreasing children’s MD mechanisms, as well as their reported victimization [97]. Results from these investigations and our own results highlight the need to further investigate the relationship between MD and empathy, both specifically in cyberbullying situations and how these relatively new constructs may be integrated and managed in anti-cyberbullying intervention.

### Limitations and future directions

This study is not without limitations, and some important suggestions can be addressed in future research. One important limitation is the fact that a convenience sample was used, and a relatively small sample size was utilized; therefore, results should be considered with caution. Further studies could include greater sample sizes from diverse countries.

Considering the design, for technical reasons, the OPT2BGood program included the four sessions of the serious game Com@Viver, and after these sessions, the four gamified tasks occurred. With this type of structure, when participants were called to reflect about the game sessions, their performance in the game along with their recollections of their cyberbullying scenario may not be fully accurate. In order to narrow this gap, at the beginning of each reflection session, participants were reminded of their performance in key parts of the game, as well as the cyberbullying event. Nonetheless, in future studies, the implementation of the OPT2BGood program should be modified and following each game session. In other words, each session should correspond to its gamified task.

Moreover, despite the study design being longitudinal as it included 4 time periods, it is important to highlight that the four sessions have only one week of interval between them. Therefore, in future investigations it would be important to test a longitudinal design with a greater time difference between sessions.

Furthermore, research has shown that the lack of significant results regarding long-term effectiveness of anti-bullying and anti-cyberbullying programs, highlights the importance of maintaining these programs [11]. This may be achieved by designing follow-up sessions, where participants could be reminded of the ICT specificities that facilitate cyberbullying, the consequences for victims, along with the need to control the automatism of psychological mechanisms, such as MD mechanisms. Furthermore, the design of this investigation did not allow us to see if the changes in MD, would translate into behavioral changes of participants [98], if they witnessed real cyberbullying events. Therefore, it would be interesting to evaluate the intentions to help, considering that the intentions are the best predictor of action [99], and that empathy increases the strength of the intentions to help [100].

In addition, the use of different MD mechanisms may have a socio-cultural foundation [64], therefore results must be carefully considered, and further studies should be conducted with different countries, in order to compare results.

Lastly, neither the sex of those involved in the cyberbullying situations, nor the ingroup and outgroup, were controlled for in the statistical analysis in this study. However, we attempted to counterbalance these features when designing the scenarios. Nonetheless, it would be important to understand if by controlling these variables the results would vary. Hence, future research could focus on controlling these variables in longitudinal analyses. Despite the fact that the results from the participants who played the serious games and the gamified tasks (experimental group) were the only ones analyzed in this study, it would be important in future studies to compare their results with a control group, in order to understand if the changes would also occur in this group or if the trends presented in this study would be different.

### Theoretical pertinence and implications for practice

This work brought an important contribution, as it analyzed the role between MD and empathy, in digital gamified tasks about a serious game. As suggested in the literature [5], a MD activity tailored to cyberbullying situations was used in this study, as several authors argue that it is necessary for interventions, since it might provide stronger effects [96]. Moreover, the activities analyzed here are an important part of interventions pertaining to serious games, as they alone allow both in-game debriefing, as well as end-of-game debriefing, which in turn, will impact simulation into a real contribution to people’s lives [72]. Our theoretical framework is detailed and well established, as some authors consider extremely important for both school-based prevention and intervention cyberbullying programs [101]. Lastly, our investigation demonstrates that improving moral values and empathy, may also be used to help bystanders act more prosocially, and are not specific elements for cyberbullies [37].

## Conclusion

The innovation of this study lies in how a gamified task about MD, tailored to cyberbullying scenarios, could serve as a mean for intervention, considering that it was based on participants’ perspectives about the phenomenon and the power that debriefing might have in these types of activities [72]. Ultimately, as recommended in the literature, these digital activities provide students a safe environment, where they could reflect about the cyberbullying incidents [9] they witnessed when playing the serious game Com@Viver [6, 7], as well as the choices they made, about how to deal with those incidents. Moreover, the gamified tasks allowed participants, to experience the consequences of being prosocial, since they receive a message from the victim, thanking them for their support. Thus, by receiving this type of social reward, the cognitive costs of feeling empathy, might decrease [49], which could lead to higher feelings of empathy, and consequently, lower levels of MD. Finally, we examined how with these gamified tasks, participants may be able get a deeper understanding of how much their support can be important in real life situations, how they can act prosocially, and how they can have a positive impact by setting the example in their peer group.

### Electronic supplementary material

Below is the link to the electronic supplementary material.

Supplementary Material 1

## Data Availability

The datasets presented in this article are not readily available because the Portuguese National Commission of Data Protection and the Deontology Committee of the researchers’ institution do not allow the availability of the datasets. The data that supports the findings of this study are available in the Supplementary material of this article.

## References

[1] Scheithauer H, Schultze-Krumbholz A, Pfetsch J, Hess M, Peter SK, Norman JO (2021). Types/Forms of Cyberbullying. The Wiley-Blackwell handbook of bullying: a Comprehensive and International Review of Research and Intervention.

[2] Francisco SM. The role of moral disengagement in cyberbullying. PhD [Dissertation]. Lisboa: Faculdade de Psicologia da Universidade de Lisboa; 2022.

[3] Allison KR, Bussey K (2016). Cyber-bystanding in context: a review of the literature on witnesses’ responses to cyberbullying. Child Youth Serv Rev.

[4] Van Cleemput K, Vandebosch H, Pabian S (2014). Personal characteristics and contextual factors that determine helping, joining in, and doing nothing when witnessing cyberbullying. Aggress Behav.

[5] DeSmet A, Bastiaensens S, Van Cleemput K, Poels K, Vandebosch H, Deboutte G, Herrewijn L, Malliet S, Pabian S, Van Broeckhoven F, De Troyer O, Deglorie G, Van Hoecke S, Samyn K, De Bourdeaudhuij I (2018). The efficacy of the friendly Attac serious digital game to promote prosocial bystander behavior in cyberbullying among young adolescents: a cluster-randomized controlled trial. Comput Hum Behav.

[6] Ferreira PC, Veiga Simão AM, Paiva A, Martinho C, Prada R, Ferreira A, Santos F (2021). Exploring empathy in cyberbullying with serious games. Comput Educ.

[7] Ferreira PC, Veiga Simão AM, Paiva A, Martinho C, Prada R, Rocha J (2022). Serious game-based psychosocial intervention to foster prosociality in cyberbullying bystanders. Psychos Interv.

[8] Moreton L, Greenfield S. University students’ views on the impact of Instagram on mental wellbeing: a qualitative study. BMC Psychol. 2022;10(45). 10.1186/s40359-022-00743-6. 10.1186/s40359-022-00743-6 PMC8883692 35227331

[9] Calvo-Morata A, Alonso-Fernández C, Freire-Moran M, Martínez-Ortiz I, Fernandez-Manjón B (2020). Serious games to prevent and detect bullying and cyberbullying: a systematic serious games and literature review. Comp Educ.

[10] Nocentini A, Zambuto V, Menesini E (2015). Anti-bullying programs and Information and Communication Technologies (ICTs): a systematic review. Aggress Violent Behav.

[11] Falla D, Romera E, Ortega-Ruiz R (2021). Aggression, moral disengagement and empathy: a longitudinal study within the interpersonal dynamics of bullying. Front Psychol.

[12] Marín-López I, Zych I, Ortega-Ruiz R, Monks CP, Llorent VJ (2020). Empathy online and moral disengagement through technology as longitudinal predictors of cyberbullying victimization and perpetration. Child Youth Serv Rev.

[13] Ng ED, Chua JYX, Shorey S (2022). The effectiveness of educational interventions on traditional bullying and cyberbullying among adolescents: a systematic review and meta-analysis. Trauma Violence Abuse.

[14] Bussey K, Luo A, Fitzpatrick S, Allison K (2020). Defending victims of cyberbullying: the role of self-efficacy and moral disengagement. J Sch Psychol.

[15] Macháčková H, Dedkova L, Sevcikova A, Cerna A (2016). Bystanders’ supportive and passive responses to cyberaggression. J Sch Violence.

[16] DeSmet A, Bastiaensens S, Van Cleemput K, Poels K, Vandebosch H, Cardon G, De Bourdeaudhuij I (2016). Deciding whether to look after them, to like it, or leave it: a multidimensional analysis of predictors of positive and negative bystander behaviour in cyberbullying among adolescents. Comput Hum Behav.

[17] Sarmiento A, Herrera-López M, Zych I (2019). Is cyberbullying a group process? Online and offline bystanders of cyberbullying act as defenders, reinforcers and outsiders. Comput Hum Behav.

[18] DeSmet A, Veldeman C, Poels K, Bastiaensens S, Van Cleemput K, Vandebosch H, De Bourdeaudhuij I (2014). Determinants of self-reported bystander behavior. Cyberpsychol Behav Soc Netw.

[19] Bastiaensens S, Vandebosch H, Poels K, Van Cleemput K, DeSmet A, De Bourdeaudhuij I (2014). Cyberbullying on social network sites: an experimental study into bystanders’ behavioural intentions to help the victim or reinforce the bully. Comput Hum Behav.

[20] Kozubal M, Szuster A, Barlińska J (2019). Cyberbystanders, affective empathy and social norms. Stud Psychol.

[21] Latané B, Darley JM (1970). Theunresponsive bystander: why doesn’t he help?.

[22] Macháčková H, Dedkova L, Mezulanikova K (2015). Brief report: the bystander effect in cyberbullying incidents. J Adolesc.

[23] Bandura A (1986). Social foundations of thought and action: a social cognitive theory.

[24] Kowalski RM, Giumetti GW, Schroeder AN, Lattanner MR (2014). Bullying in the digital age: a critical review and meta-analysis of cyberbullying research among youth. Psychol Bull.

[25] Pabian S, Vandebosch H, Poels K, Van Cleemput K, Bastiaensens S (2016). Exposure to cyberbullying as a bystander: an investigation of desensitization effects among early adolescents. Comput Hum Behav.

[26] Hswen Y, Rubenzahl L, Bickham DS (2014). Feasibility of an online and Mobile Videogame Curriculum for Teaching Children Safe and Healthy Cellphone and internet behaviors. Games Health J.

[27] De Troyer O, Helalouch A, Debruyne C. Towards Computer-Supported Self-debriefing of a Serious Game Against Cyber Bullying. In: Bottino R, Jeuring J, Veltkamp RC, eds. Games and Learning Alliance: 5th International Conference, GALA 2016, Utrecht, The Netherlands, December 5–7, 2016, Proceedings. Lecture Notes in Computer Science. Vol. 10056. Springer International Publishing; 2016;374–384.10.1007/978-3-319-50182-6_34.

[28] Revuelta Domínguez FI, Guerra-Antequera J, Antequera-Barroso JA, Pedrera (2023). -Rodríguez, M. I. exploring the impact of the video game Monité on exogenous factors and resilience against bullying in primary education students. Educ Sci.

[29] DeSmet A, Bastiaensens S, Van Cleemput K, Poels K, Vandebosch H, Deboutte G, Herrewijn L, Malliet S, Pabian S, Van Broeckhoven F, De Troyer O, Deglorie G, Van Hoecke S, Samyn K, De Bourdeaudhuij I (2018). The efficacy of the Friendly Attac serious digital game to promote prosocial bystander behavior in cyberbullying among young adolescents: A cluster-randomized controlled trial. Comput Hum Behav.

[30] Garaigordobil M, Martínez-Valderrey V (2018). Technological resources to prevent cyberbullying during adolescence: the Cyberprogram 2.0 program and the cooperative Cybereduca 2.0 Videogame. Front Psychol.

[31] Morata AC, Morán MF, Ortiz IM, Manjón BF. Conectado: A serious game to raise awareness of bullying and cyberbullying in high schools. 2018.

[32] Mikka-Muntuumo J, Peters A, Jazri H. CyberBullet-Share Your Story: an interactive game for stimulating awareness on the harm and negative effects of the internet. In: Proceedings of the Second African Conference for Human Computer Interaction: Thriving Communities. 2018, December, pp. 1–4. 10.1145/3283458.3283482.

[33] Lazarinis F, Alexandri K, Panagiotakopoulos C, Verykios VS (2020). Sensitizing young children on internet addiction and online safety risks through storytelling in a mobile application. Educ Inf Technol.

[34] Bastiaensens S, Pabian S, Vandebosch H, Poels K, Van Cleemput K, DeSmet A, De Bourdeaudhuij I (2016). From normative influence to social pressure: how relevant others affect whether bystanders join in cyberbullying. Soc Dev.

[35] Barlińska J, Szuster A, Winiewski M (2018). Cyberbullying among adolescent bystanders: role of affective versus cognitive empathy in increasing prosocial cyberbystander behavior. Front Psychol.

[36] Marín-López I, Zych I, Ortega-Ruiz R, Monks C (2019). Validación Y propiedadespsicométricas del cuestionario de Empatía online y elCuestionario De Desconexión Moral a través de las Tecnologías.[Validation and psychometric properties of the Online Empathy Questionnaire and the Moral Disengagement through technologies Questionnaire]. Creando Redes Doctorales.

[37] Macháčková H (2020). Bystander reactions to cyberbullying and cyberaggression: individual, contextual, and social factors. Curr Opin Psychol.

[38] Bandura A (2002). Selective moral disengagement in the exercise of moral agency. J Moral Educ.

[39] Bandura A, Barbaranelli C, Caprara GV, Pastorelli C (1996). Mechanisms of moral disengagement in the exercise of moral agency. J Pers Soc Psychol.

[40] Bandura A (2001). Social Cognitive Theory: an agentic perspective. Annu Rev Psychol.

[41] Baldry AC, Farrington DP, Sorrentino A, Am (2015). I at risk of cyberbullying? A narrative review and conceptual framework for research on risk of cyberbullying and cybervictimization: the risk and needs assessment approach. Aggress Violent Behav.

[42] Chen L, Ho SS, Lwin MO (2017). A meta-analysis of factors predicting cyberbullying perpetration and victimization: from the social cognitive and media effects approach. New Media Soc.

[43] Guo S (2016). A meta-analysis of the predictors of cyberbullying perpetration and victimization. Psychol Sch.

[44] Luo A, Bussey K (2022). Mediating role of moral disengagement in the perpetration of cyberbullying by victims and bystanders. J Adolesc.

[45] Knauf RK, Eschenbeck H, Hock M. Bystanders of bullying: social-cognitive and affective reactions to school bullying and cyberbullying. Cyberpsychol: J Psychos Res Cyberspace. 2018;12(4). 10.5817/CP2018-4-3.

[46] Wachs S (2012). Moral disengagement and emotional and social difficulties in bullying and cyberbullying: differences by participant role. Emot Behav Diff.

[47] Lazuras L, Pyżalski J, Barkoukis V, Tsorbatzoudis H (2012). Empathy and moral disengagement in adolescent cyberbullying: implications for educational intervention and pedagogical practice. Stud Eduk.

[48] Price D, Green D, Spears B, Scrimgeour M, Barnes A, Geer R, Johnson B (2014). A qualitative exploration of cyber-bystanders and moral engagement. Aust J Guid Couns.

[49] Jeong R, Gilbertson M, Riffle LN, Demaray MK (2022). Participant role behavior in cyberbullying: an examination of moral disengagement among college students. Int J Bullying Prev.

[50] Leduc K, Conway L, Gomez-Garibello C, Talwar V (2018). The influence of participant role, gender, and age in elementary and high-school children’s moral justifications of cyberbullying behaviors. Comput Hum Behav.

[51] Raboteg-Šarić Z, Bartaković S (2019). Empathy and Moral Disengagement as predictors of Bystander roles in School Bullying.Cent Eur. J Paedtrics.

[52] Thornberg R. Longitudinal link between moral disengagement and bullying among children and adolescents: a systematic review. Eur J Dev Psychol. 2023;1–31. 10.1080/17405629.2023.2191945.

[53] Bjärehed M, Thornberg R, Wänström L, Gini G (2020). Mechanisms of moral disengagement and their associations with indirect bullying, direct bullying, and pro-aggressive bystander behavior. J Early Adolesc.

[54] Coll MP, Viding E, Rütgen M, Silani G, Lamm C, Catmur C, Bird G (2017). Are we really measuring empathy? Proposal for a new measurement framework. Neurosci Biobehav Rev.

[55] Cameron CD, Hutcherson CA, Ferguson AM, Scheffer JA, Hadjiandreou E, Inzlicht M (2019). Empathy is hard work: people choose to avoid empathy because of its cognitive costs. J Exp Psychol Gen.

[56] Hall JA, Schwartz R (2019). Empathy present and future. J Soc Psychol.

[57] Batson CD. These things called empathy: Eight related but distinct phenomena. In: Decety J, Ickes W, eds. The Social Neuroscience of Empathy. Boston Review; 2009. pp. 3–15. 10.7551/mitpress/9780262012973.003.0002.

[58] Hoffman ML. Empathy, social cognition, and moral action. In: Killen M, Smetana JG, editors. Handbook of Moral Behavior and Development. Psychology; 2014. pp. 299–326.

[59] Baron-Cohen S, Wheelwright S (2004). The Empathy Quotient: an investigation of adults with Asperger syndrome or high functioning autism, and normal sex differences. J Aut Dev Disord.

[60] Cuff BMP, Brown SJ, Taylor L, Howat DJ (2014). Empathy: a review of the concept. Emot Rev.

[61] Barlińska J, Szuster A, Winiewski M (2013). Cyberbullying among adolescent bystanders: role of the communication medium, form of violence, and empathy. J Community Appl Soc Psychol.

[62] Hodges SD, Myers MW, Roy FB, Kathleen DV (2007). Empathy. Encyclopedia of social psychology.

[63] Zhu C, Huang S, Evans R, Zhang W (2021). Cyberbullying among adolescents and children: a comprehensive review of the global situation, risk factors, and preventive measures. Front Public Health.

[64] Wang S (2021). Standing up or standing by: Bystander intervention in cyberbullying on social media. New Media Soc.

[65] Huang L, Li W, Xu Z, Sun H, Ai D, Hu Y, Wang S, Li Y, Zhou Y (2023). The severity of cyberbullying affects bystander intervention among college students: the roles of feelings of responsibility and empathy. Psychol Res Behav Manag.

[66] Francisco SM, Ferreira PC, Veiga Simão AM, Pereira NS. Measuring empathy online and moral disengagement in cyberbullying. Front Psychol. 2023;14. 10.3389/fpsyg.2023.1061482 10.3389/fpsyg.2023.1061482 PMC10172580 37179897

[67] Paciello M, Fida R, Cerniglia L, Tramontano C, Cole E (2013). High cost helping scenario: the role of empathy, prosocial reasoning and moral disengagement on helping behavior. Pers Individ Dif.

[68] Shen Y, Yuan L, Xiong X, Xin T (2023). Empathy and cyberbystander behavior: the role of moral disengagement. Curr Psychol.

[69] Palladino BE, Nocentini A, Menesini E (2016). Evidence-based intervention against bullying and cyberbullying: evaluation of the NoTrap! Program in two independent trials. Aggress Behav.

[70] Haddock AD, Jimerson SR (2017). An examination of differences in moral disengagement and empathy among bullying participant groups. J Relationships Res.

[71] Zych I, Llorent VJ (2019). Affective empathy and moral disengagement related to late adolescent bullying perpetration. Ethics Behav.

[72] Brewer G, Kerslake J (2015). Cyberbullying, self-esteem, empathy and loneliness. Computn Hum Behav.

[73] Francisco SM, Ferreira PC, Veiga Simão AM. Behind the scenes of cyberbullying: personal and normative beliefs across profiles and moral disengagement mechanisms. Int J Adolesc Youth. 2022;27(1):337–61. 10.1080/02673843.2022.2095215.

[74] De Troyer O, Helalouch A, Debruyne C. Towards computer-supported self-debriefing of a serious game against cyberbullying. In: Bottino R, Jeuring J, Veltkamp RC, eds. Games and Learning Alliance: 5th International Conference, GALA 2016, Utrecht, The Netherlands, December 5–7, 2016, Proceedings. Springer International Publishing; 2016. pp. 374–384. 10.1007/978-3-319-50182-6_34.

[75] O’Keeffe GS, Clarke-Pearson K (2011). On communications and media. Clinical report-the impact of social media on children, adolescents, and families. J Am Acad Pediatr.

[76] Antunes DMP, Com@Viver. Using affective AI agents to encourage prosocial activity. Master Thesis. Instituto Superior Técnico; 2020. Available at https://fenix.tecnico.ulisboa.pt/cursos/meic-t/dissertacao/1691203502344026.

[77] Veiga Simão AM, Ferreira P, Francisco SM, Paulino P, Souza SB, Cyberbullying (2018). Shaping the use of verbal aggression through normative moral beliefs and self-efficacy. New Media Soc.

[78] Veiga Simão AM, Ferreira PC, Pereira N, Oliveira S, Com@viver. Promover comportamentos pró-sociais. Investigação e intervenção no âmbito do cyberbullying. In: Michelon FF, ed. A Universidade do Encontro e Inclusão. Conferência e Mesas da 4ª SIIEPE. Brasil: Universidade Federal de Pelotas; 2019. pp. 36–47. ISBN: 978-85-517-0036-5.

[79] Fanning RM, Gaba DM (2007). The role of debriefing in simulation-based learning. Simul Healthc.

[80] Crookall D (2010). Serious games, debriefing, and simulation/gaming as a discipline. Simul Gaming.

[81] Linacre JM. Winsteps®Rasch measurement computer program. Beaverton, Oregon: Winsteps.com.2019.

[82] Bond TG, Fox (2007). CM. Applying the Rasch model.

[83] Smith EV (2001). Evidence for the reliability of measures and validity of measure interpretation: a Rasch measure perspective. J Appl Meas.

[84] Fox CM, Jones JA (1998). Uses of raschmodeling in counseling psychology research. J Couns Psychol.

[85] Snijders TAB, Bosker R (1999). Multilevel analysis: an introduction to basic and advanced multilevel modeling.

[86] Heck RH, Thomas SL, Tabata LN (2012). Multilevel modeling of categorical outcomes using IBM SPSS.

[87] Singer JD, Willet JB (2003). Applied longitudinal data analysis: modeling change and event occurrence.

[88] Luo A, Bussey K (2019). The selectivity of moral disengagement in defenders of cyberbullying: contextual moral disengagement. Comput Hum Behav.

[89] Francisco SM, Veiga Simão AM, Ferreira PC, Martins MJDD, Cyberbullying (2015). The hidden side of college students. Comput Hum Behav.

[90] Staude-Müller F, Hansen B, Voss M (2012). How stressful is online victimization? Effects of victim’s personality and properties of the incident. Eur J Dev Psychol.

[91] Koehler C, Weber M. Do I really need to help?! Perceived severity of cyberbullying, victim blaming, and bystanders’ willingness to help the victim. Cyberpsychol J Psychos Res Cyberspace. 2018;12(4). 10.5817/CP2018-4-4.

[92] Tajfel H (1970). Experiments in intergroup discrimination. Sci Am.

[93] Tarrant M, Dazeley S, Cottom TM (2009). Social categorization and empathy for outgroup members. Br J Soc Psychol.

[94] Domínguez-Hernández F, Bonell L, Martínez-González A. A systematic review of factors that moderate bystanders’ actions in cyberbullying. Cyberpsychol J Psychos Res Cyberspace. 2018;12(4). 10.5817/CP2018-4-1.

[95] Huang CL, Zhang S, Yang SC (2020). How students react to different cyberbullying events: past experience, judgment, perceived seriousness, helping behavior and the effect of online disinhibition. Comput Hum Behav.

[96] Smith PK, Mahdavi J, Carvalho M, Fisher S, Russell S, Tippett N (2008). Cyberbullying: its nature and impact in secondary school pupils. J Child Psychol Psychiatry.

[97] Bussey K, Quinn Q, Dobson J (2015). The moderating role of empathic concern and perspective taking on the relationship between moral disengagement and aggression. Merrill-Palmer Q.

[98] Gini G, Pozzoli T, Hymel S (2014). Moral disengagement among children and youth: a meta-analytic review of links to aggressive behavior. Aggress Behav.

[99] Nasaescu E, Zych I, Ortega-Ruiz R, Farrington DP, Llorent VJ (2021). Stability and change in longitudinal patterns of antisocial behaviors: the role of social and emotional competencies, empathy, and morality. Curr Psychol.

[100] Cross D, Shaw T, Hadwen K, Cardoso P, Slee P, Roberts C, Thomas L, Barnes A (2016). Longitudinal impact of the Cyber Friendly schools program on adolescents’ cyberbullying behavior. Aggress Behav.

[101] Cross D, Barnes A, Papageorgiou A, Hadwen K, Hearn L, Lester L (2015). A social–ecological framework for understanding and reducing cyberbullying behaviours. Aggress Violent Behav.

[102] Barkoukis V, Lazuras L, Ourda D, Tsorbatzoudis H (2015). Tackling psychosocial risk factors for adolescent cyberbullying: evidence from a school-based intervention. Aggress Behav.

[103] Wang C, Goldberg TS (2017). Using children’s literature to decrease moral disengagement and victimization among elementary school students. Psychol Schools.

[104] Bustamante A, Chaux E (2014). Reducing moral disengagement mechanisms: a comparison of two interventions. J Latino/Latin Am Stud.

[105] Ajzen I (1991). The theory of planned behavior. Organ Behav Hum Dec Proc.

[106] Hayashi Y, Tahmasbi N (2021). Psychological predictors of bystanders’ intention to help cyberbullying victims among college students: an application of theory of planned behavior. J Interpers Violence.

[107] Tanrikulu I (2017). Cyberbullying prevention and intervention programs in schools: a systematic review. School Psychol Int.

