# Supplementary material for: Moral disengagement and empathy in cyberbullying: how they are related in reflection activities about a serious game
Source: BMC Psychol. 2024 Mar 21;12:168. doi: 10.1186/s40359-024-01582-3 (PMC10956178; doi:10.1186/s40359-024-01582-3)
Supplement: Supplementary file 1 — Supplementary Material 1 [file 40359_2024_1582_MOESM1_ESM.docx]

**Appendix A**

**Figure A1**

*Cyberbullying case number 1( image retrieved from the serious game Com@Viver)*

**
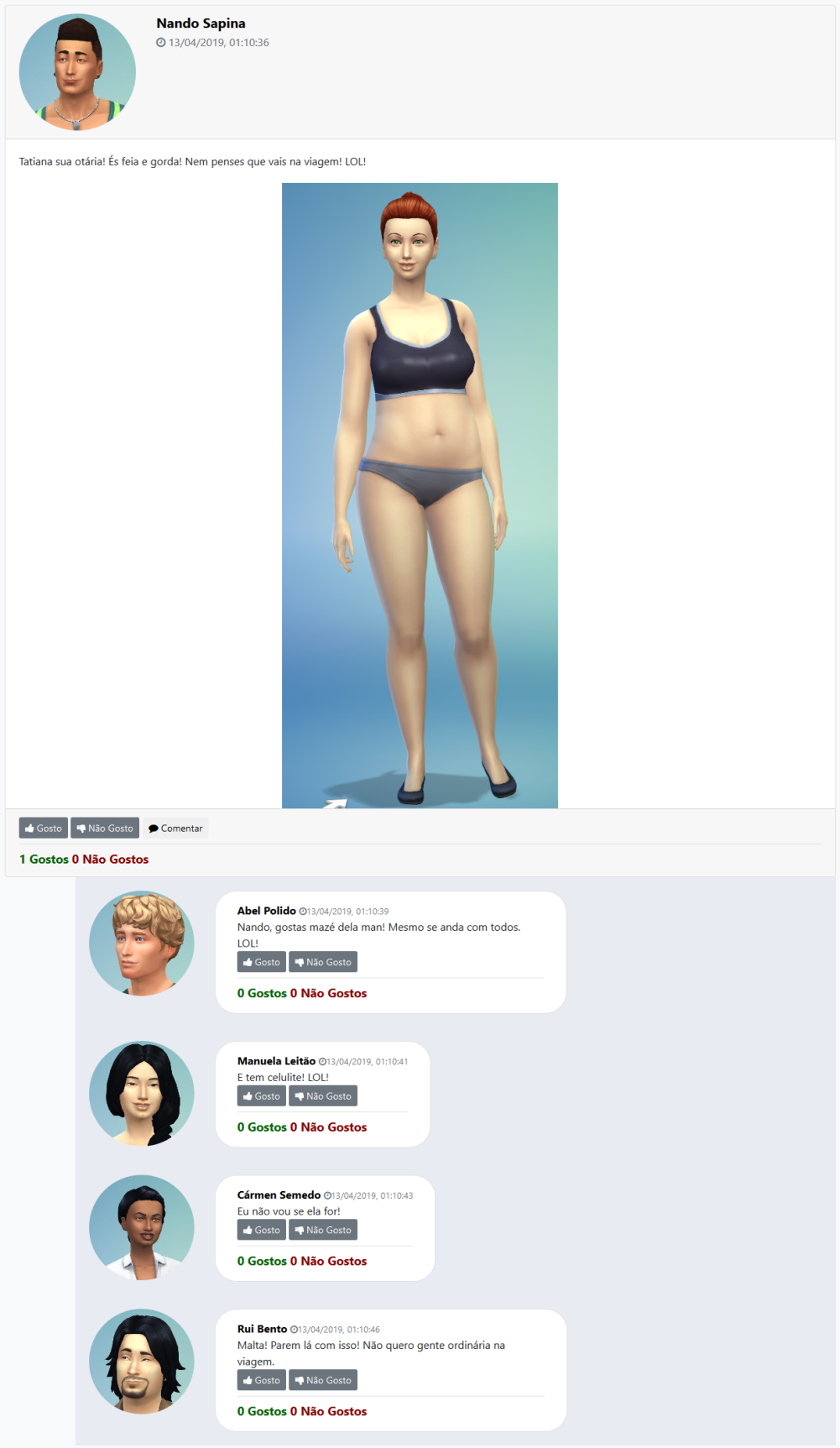
**

**Figure A2**

*Cyberbullying case number 2 (image retrieved from the serious game Com@Viver)*

**
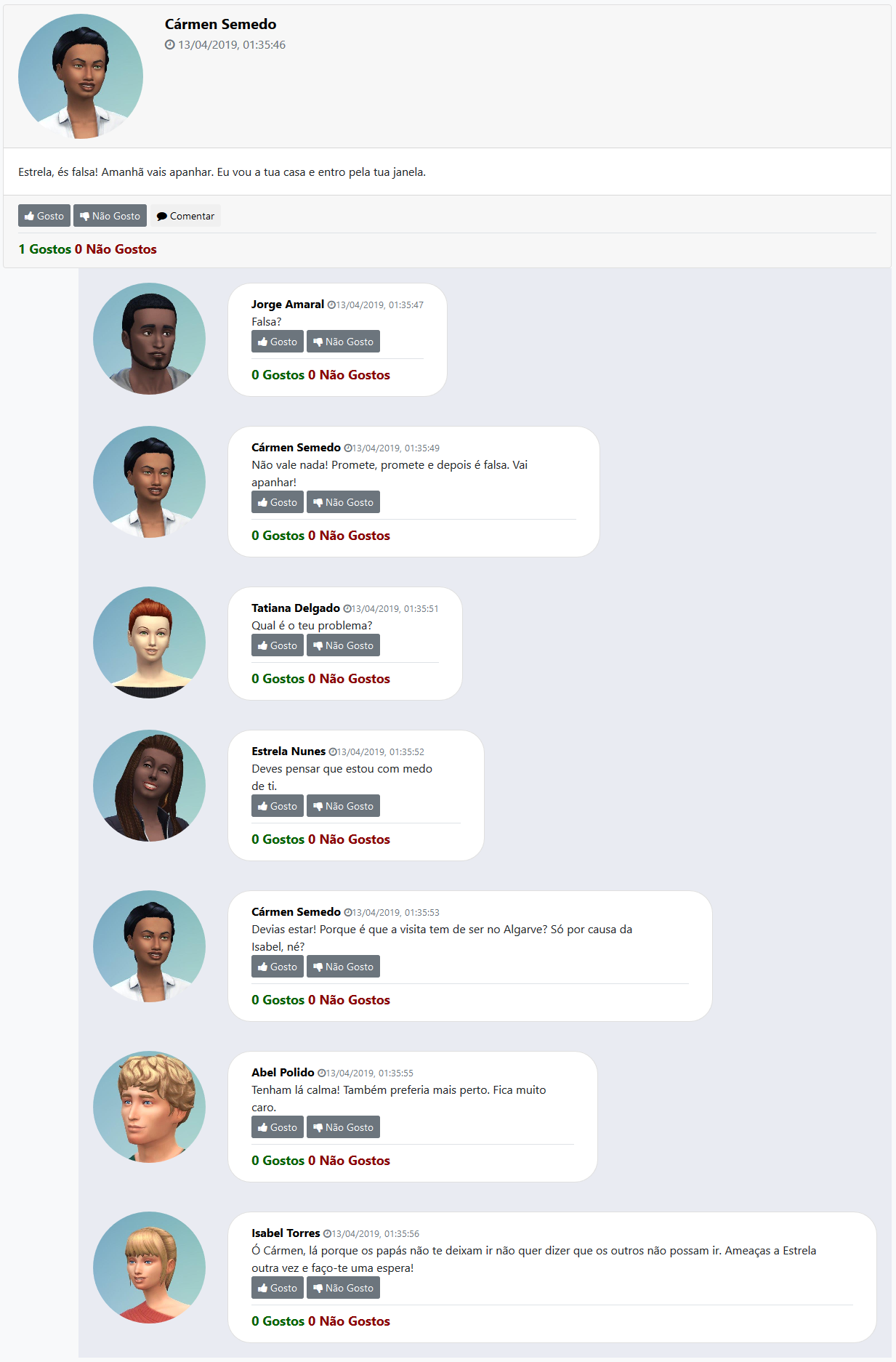
**

**Figure A3**

*Cyberbullying case number 3 (image retrieved from the serious game Com@Viver)*

**
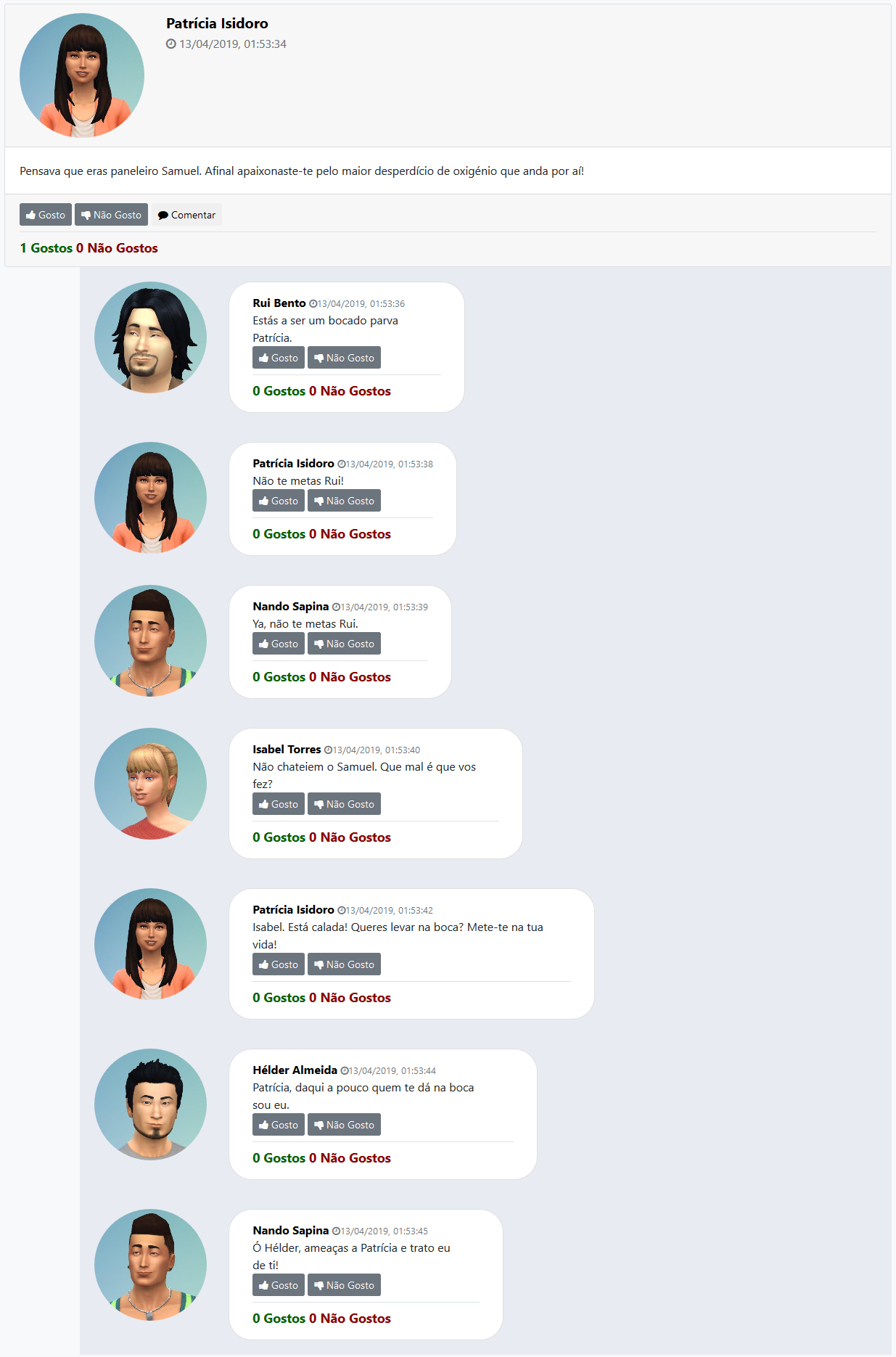
**

**Figure A4**

*Cyberbullying case number 4 (image retrieved from the serious game Com@Viver)*

**
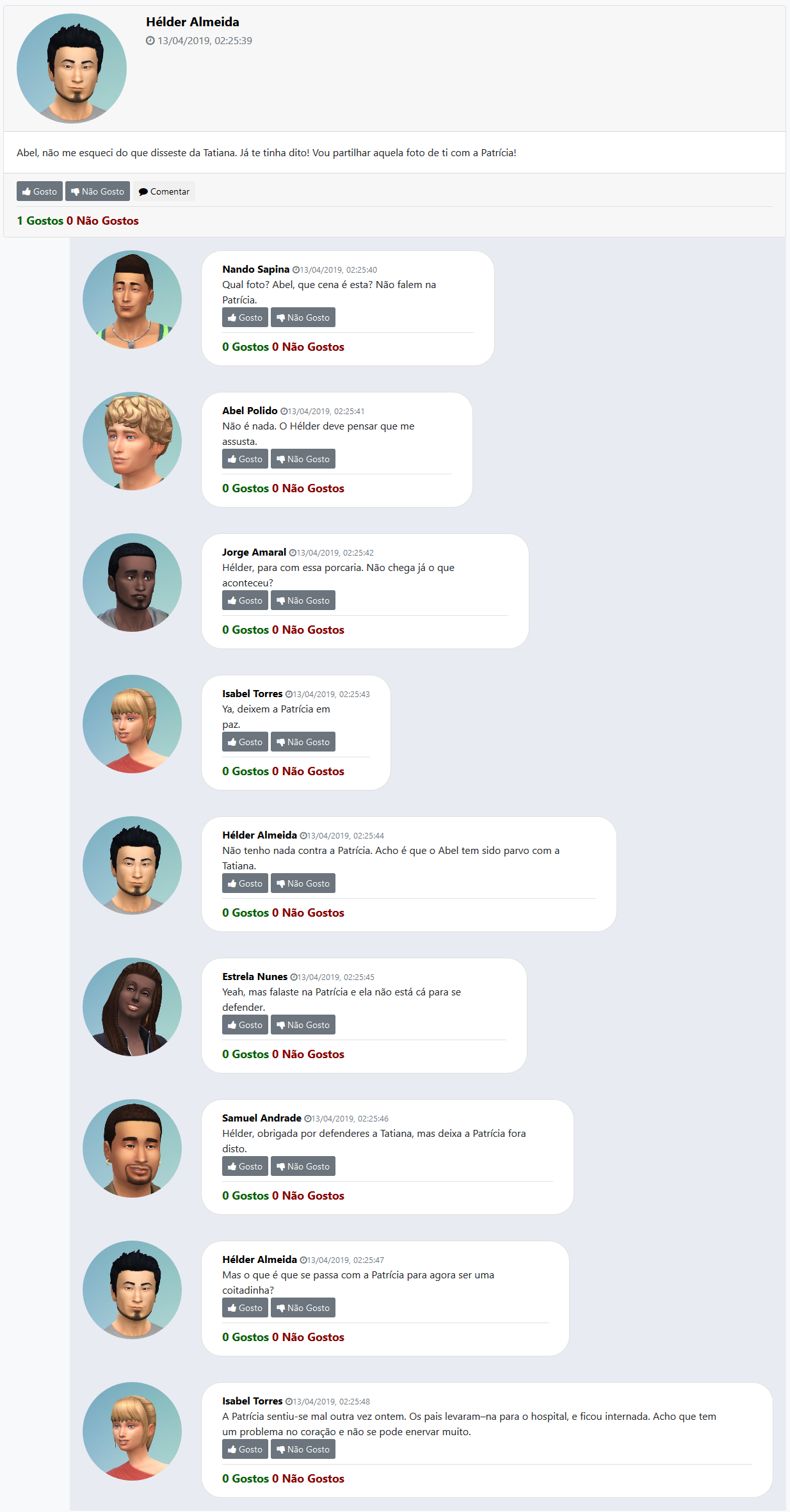
**

**Appendix B**

**Figure B1**

*Beginning of the reflection activities* *(image retrieved from the OPT2Bgood program)*

**
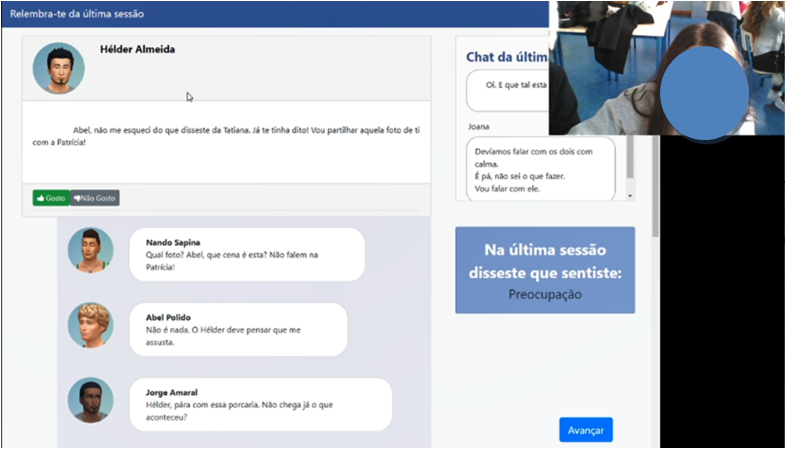
**

*Note.* The purpose of this is to help participants remember what they responded in the game. When the reflection activities initiate, the cyberbullying case appears, what bystanders have commented, liked or disliked, what emotion the player said he/she felt about the cyberbullying situation and what the player wrote in the chat when asked about what was going on.

**Figure B2**

*Moral Disengagement gamified task (image retrieved from the OPT2Bgood program)*

**
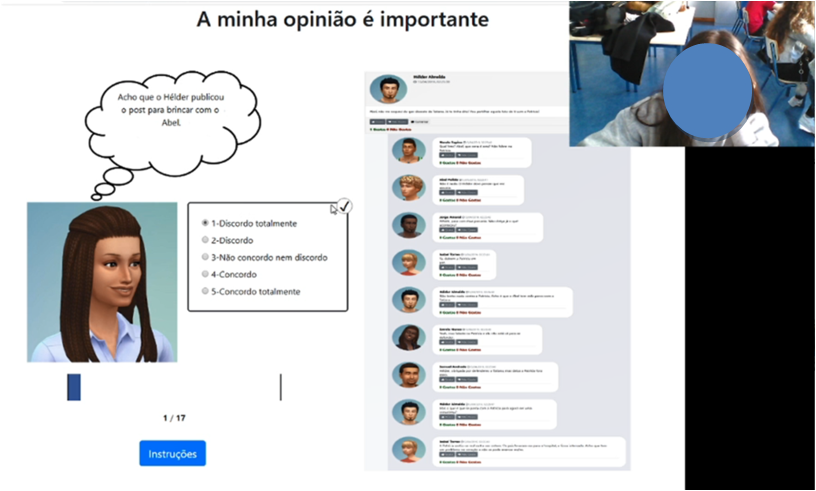
**

Note: This activity has a bar, so players can know their progress.

**Figure B3**

*In-game debriefing of the moral disengagement activity (image retrieved from the OPT2Bgood program)*

**
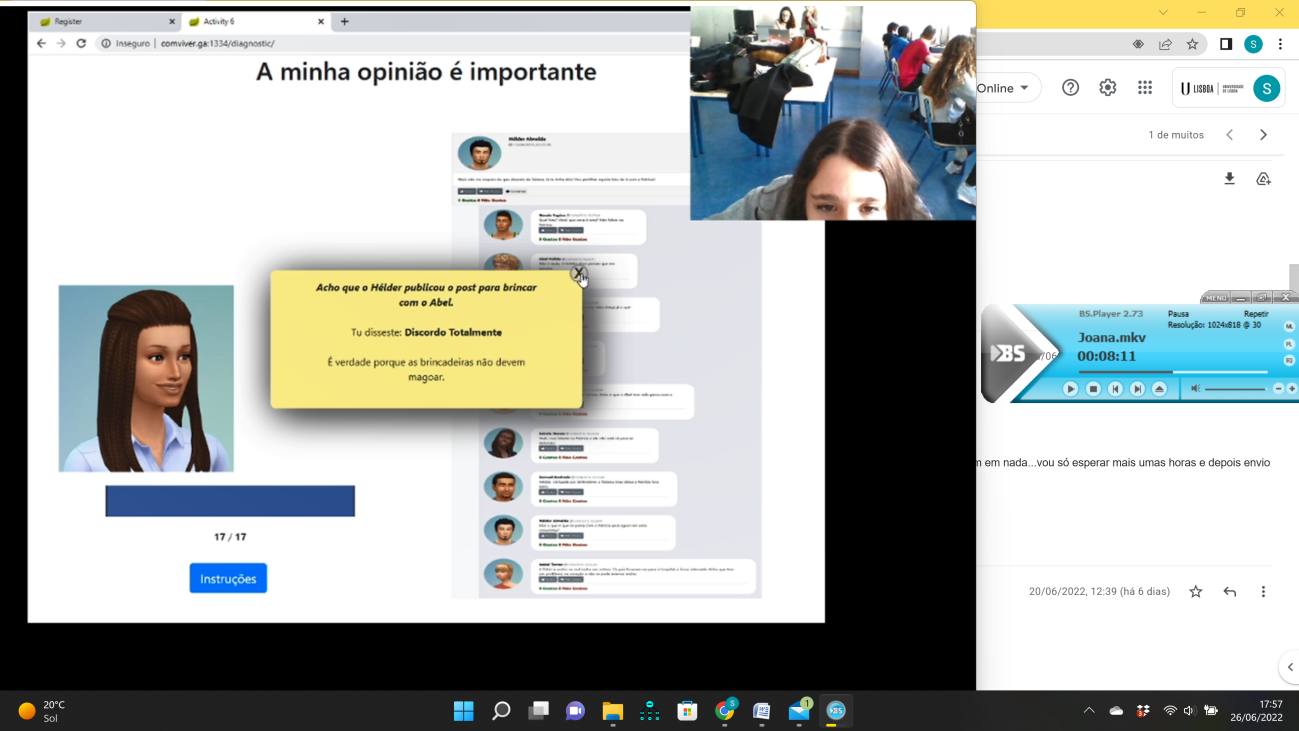
**

*Note.* This type of debriefing appears 3 times, according to 3 of the most highly or lowest coded responses, by reinforcing positive responses or by demotivating negative responses.

**Figure B4**

*
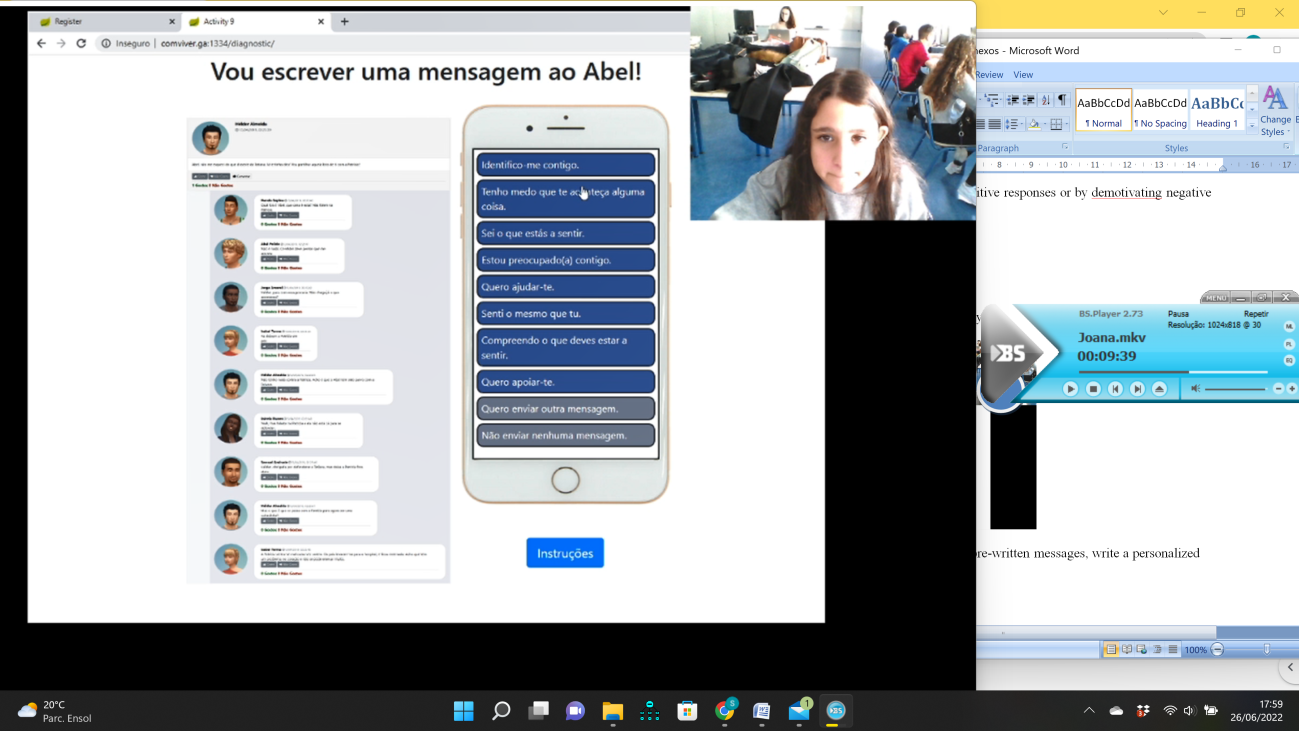
Beginning of the empathy gamified task (image retrieved from the OPT2Bgood program)*

*Note.* Participants may choose between 8 pre-written messages, write a personalized message or decide not send a message.

**Figure B5**

*During the empathy gamified task (image retrieved from the OPT2Bgood program)*

**
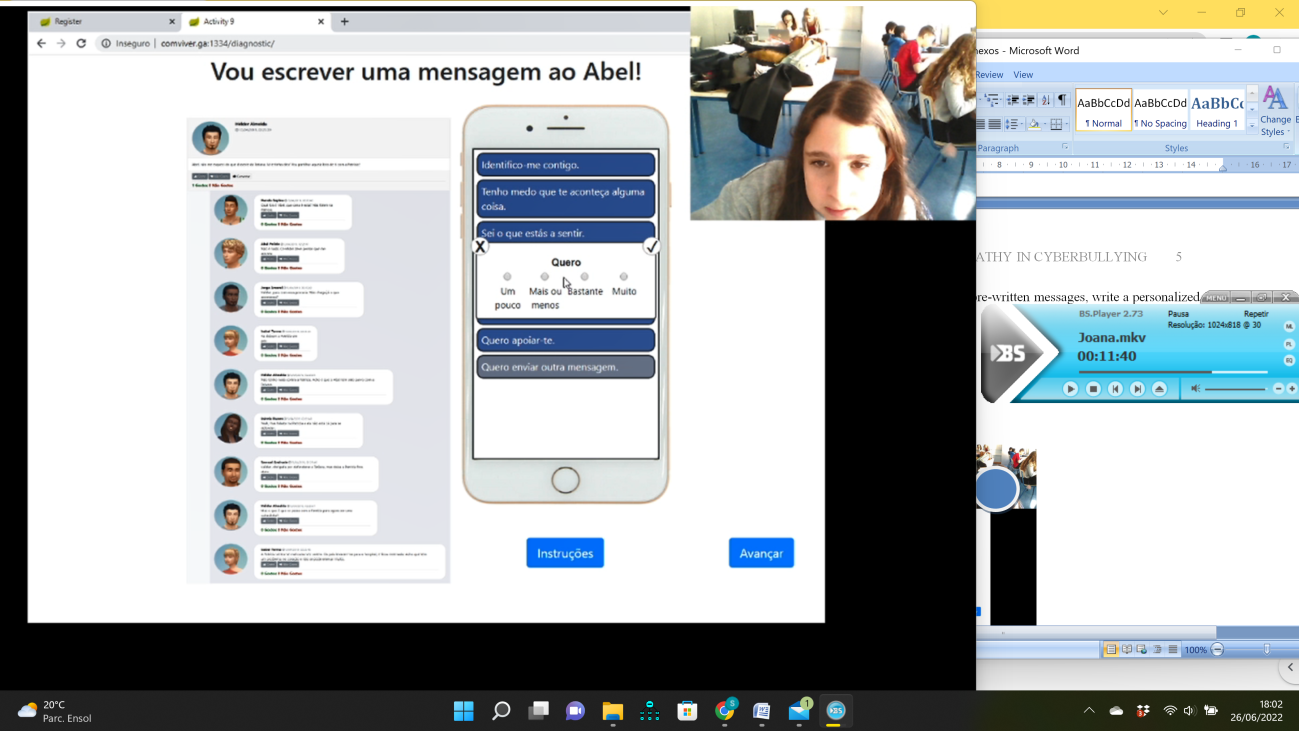
**

*Note.* When participants choose a message, he/she needs to choose between “a little” and “much”, that is, how much they agree with the selected message.

**Figure B6**

*End-game debriefing, the last rectangle presents the name of the moral disengagement gamified task (image retrieved from the OPT2Bgood program)*

**
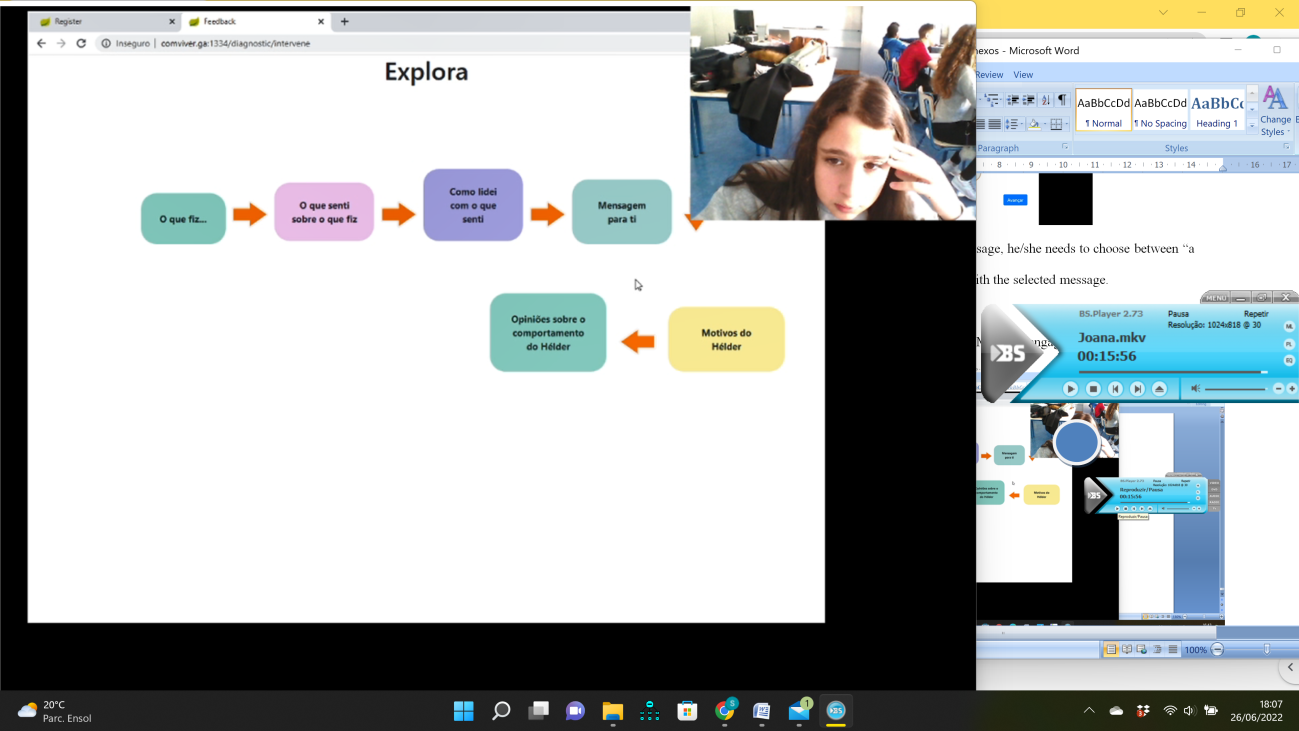
**

**Figure B7**

*End-game debriefing with the specific feedback from the moral disengagement gamified task (image retrieved from the OPT2Bgood program)*

**
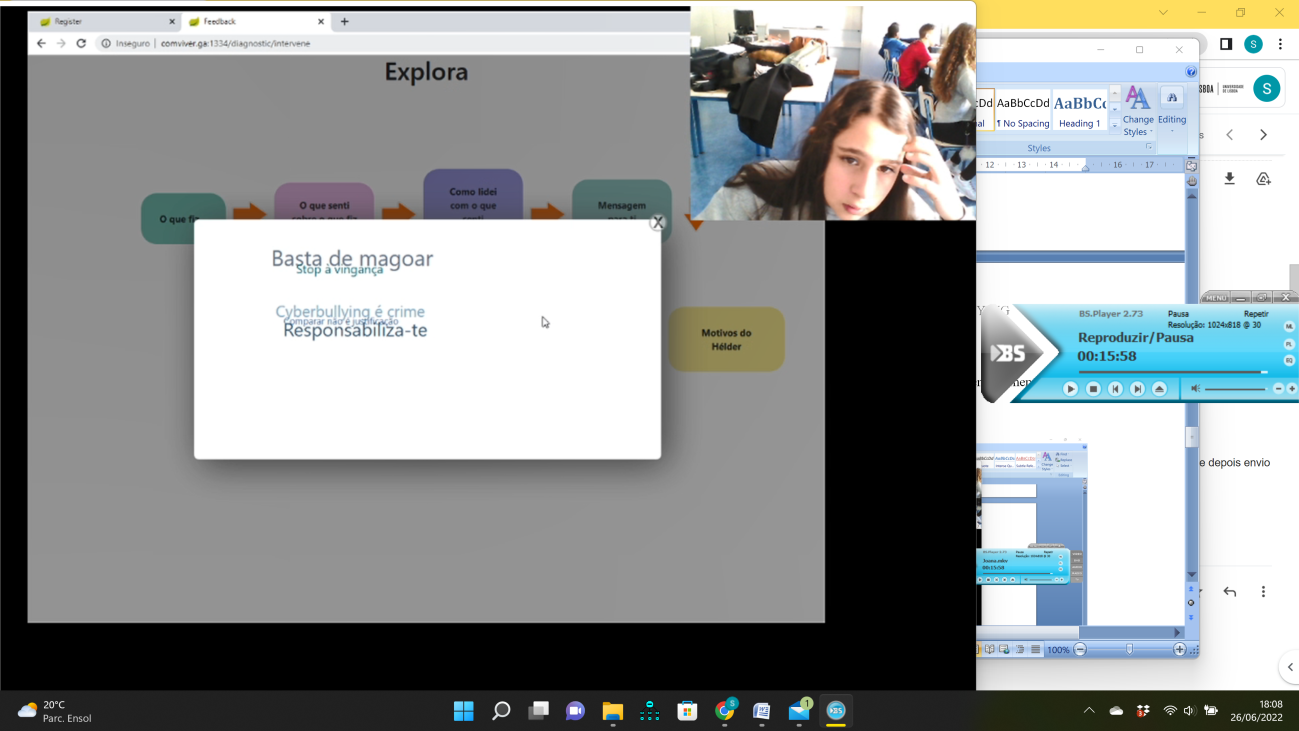
**

**Figure B8**

*End-game debriefing, the last rectangle presents the name of the empathy gamified task (image retrieved from the OPT2Bgood program)*

**
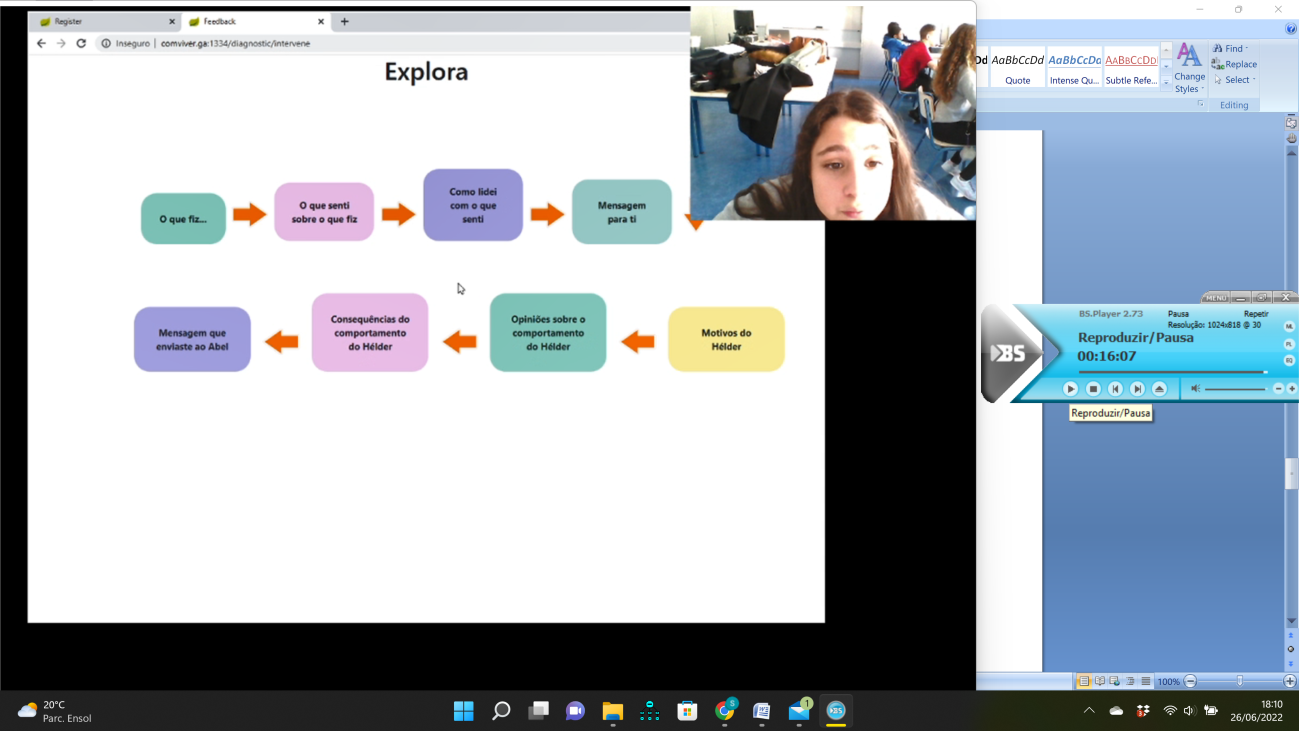
**

**Figure B9**

*End-game debriefing with the specific feedback from the empathy gamified task (image retrieved from the OPT2Bgood program)*

**
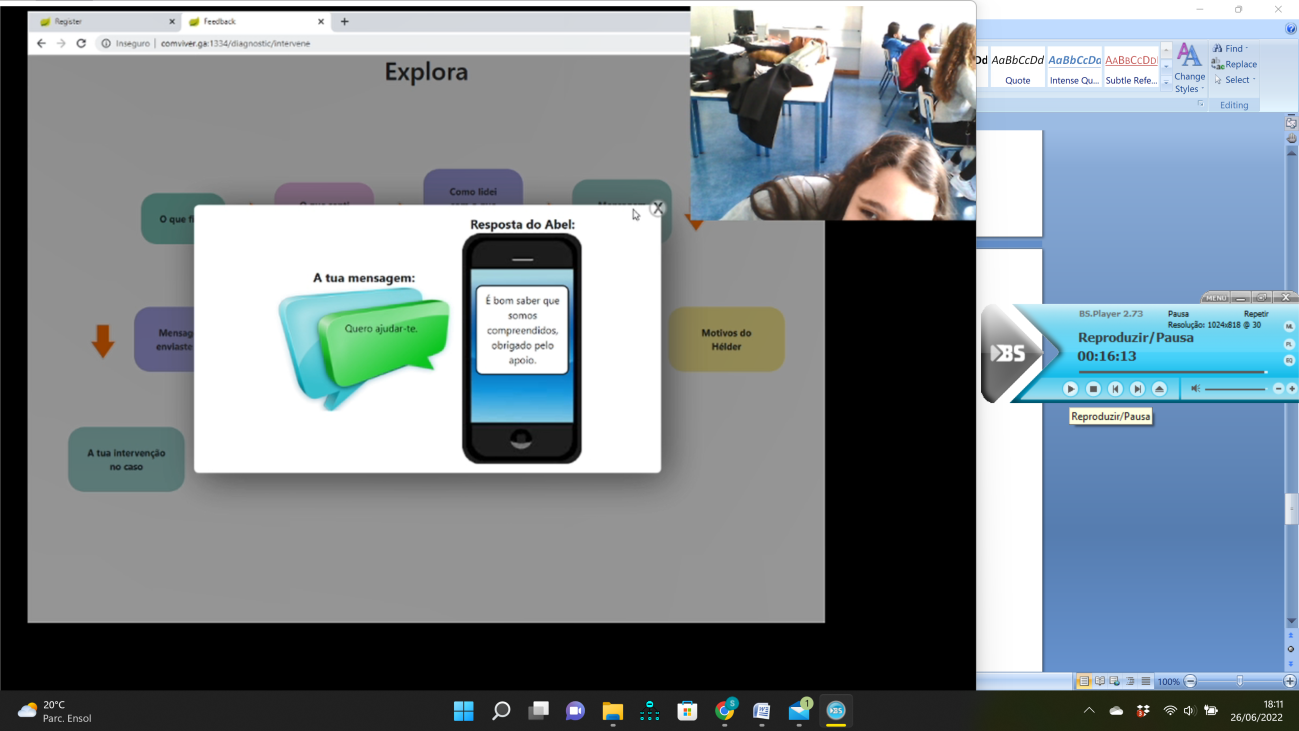
**

**Figure B10**

*End-game final debriefing with respect to participants’ intervention (image retrieved from the OPT2Bgood program)*

**
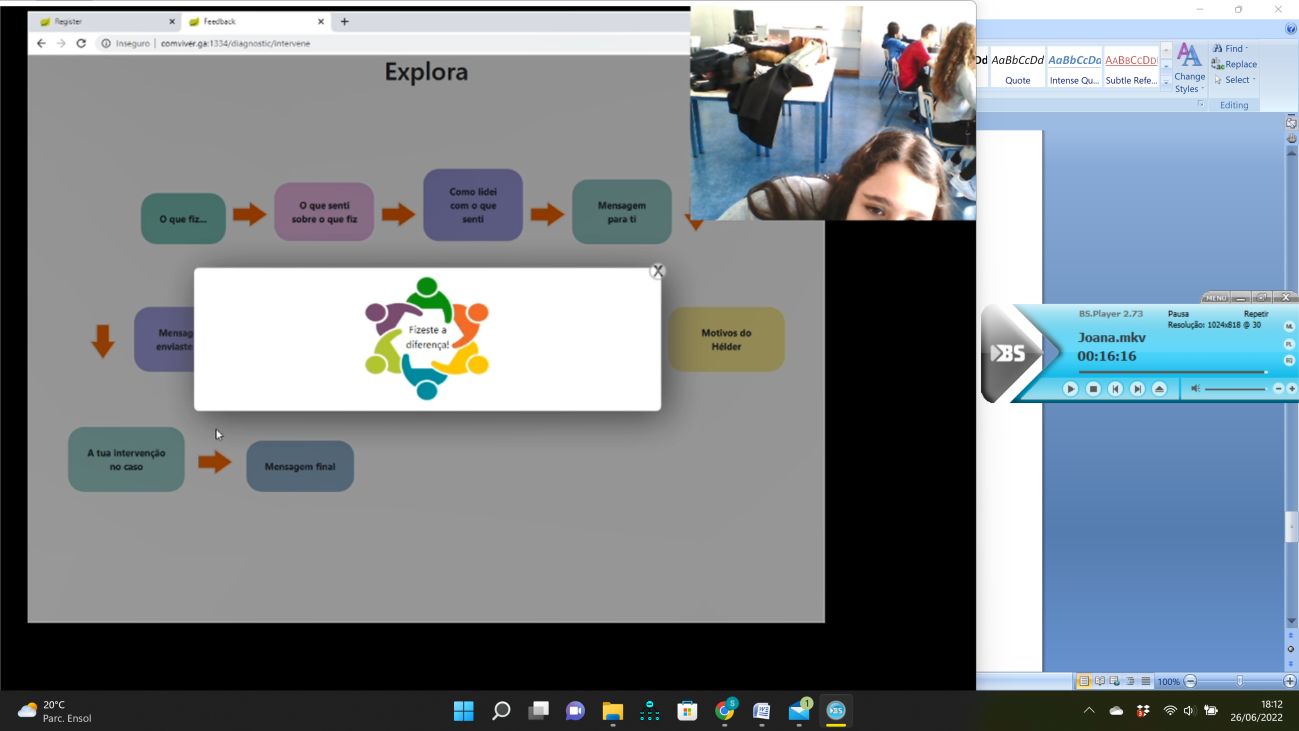
**

**Figure B11**

*End-game final debriefing with respect to the participants’ intervention, final message (image retrieved from the OPT2Bgood program)*

**
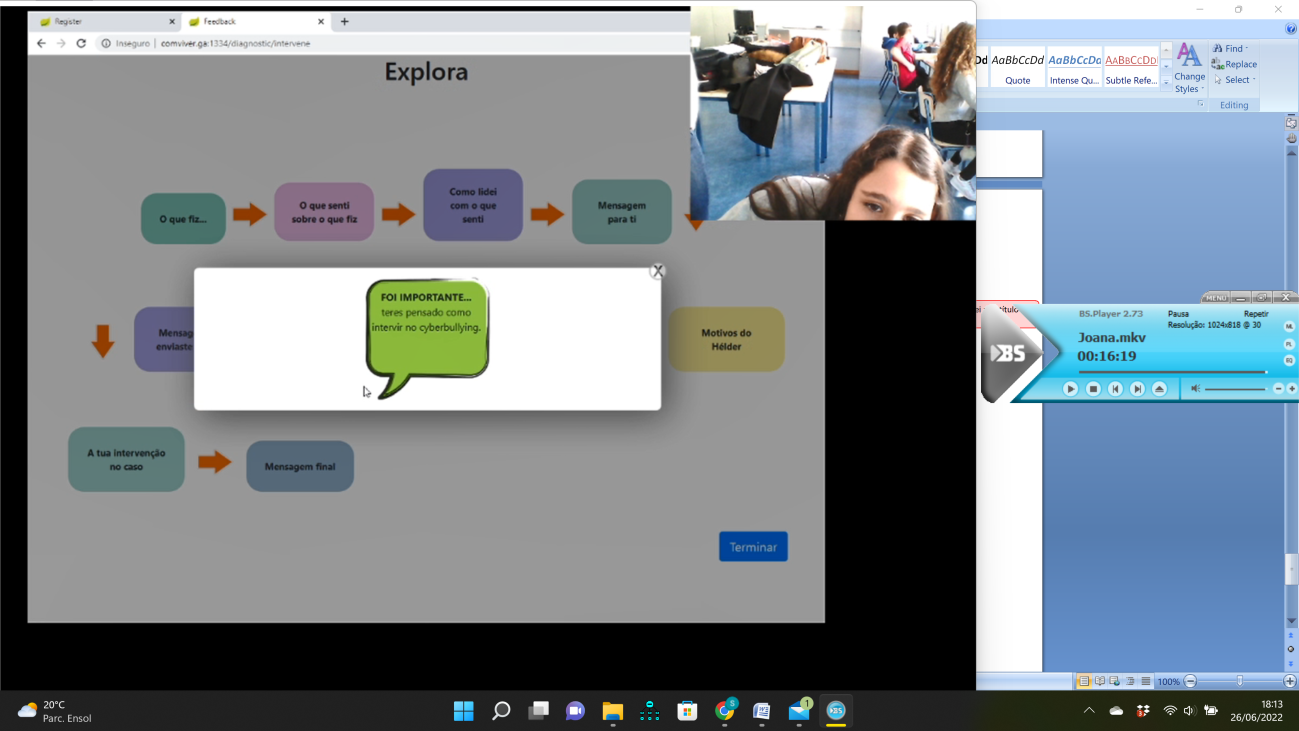
**
